# Salt Reduction Initiatives in the Eastern Mediterranean Region and Evaluation of Progress towards the 2025 Global Target: A Systematic Review

**DOI:** 10.3390/nu13082676

**Published:** 2021-07-31

**Authors:** Ayoub Al-Jawaldeh, Mandy Taktouk, Aya Chatila, Sally Naalbandian, Al-Anoud Mohammed Al-Thani, Majid M. Alkhalaf, Salima Almamary, Rawhieh Barham, Nimah M. Baqadir, Faisal F. Binsunaid, Gihan Fouad, Lara Nasreddine

**Affiliations:** 1 Regional Office for the Eastern Mediterranean (EMRO), World Health Organization (WHO), Cairo 11435, Egypt; aljawaldeha@who.int; 2 Nutrition and Food Sciences Department, Faculty of Agriculture and Food Sciences, American University of Beirut, Beirut 11-0236, Lebanon; mt86@aub.edu.lb (M.T.); aac25@mail.aub.edu (A.C.); 3 Science and Agriculture Library, American University of Beirut, Beirut 11-0236, Lebanon; sn23@aub.edu.lb; 4 Health Promotion and NCD Division, Public Health Department, Ministry of Public Health, Doha 42, Qatar; malthani@moph.gov.qa; 5 National Nutrition Committee, Saudi Food and Drug Authority, Riyadh 13312-6288, Saudi Arabia; m_khalaf75@hotmail.com (M.M.A.); nimahbaqadir@gmail.com (N.M.B.); 6 Nutrition Department, Ministry of Health, Muscat 393, Oman; dr.salima.almamary@gmail.com; 7 Nutrition Department, Ministry of Health, Amman 11118, Jordan; majeda_barham@hotmail.com; 8 Healthy Food Department, Saudi Food and Drug Authority, Riyadh 13312-6288, Saudi Arabia; ffsunaid@sfda.gov.sa; 9 National Nutrition Institute, Cairo 11435, Egypt; gihan_fouad@yahoo.com

**Keywords:** salt, reduction, strategy, implementation, evaluation, Eastern Mediterranean Region

## Abstract

This study aims at identifying national salt reduction initiatives in countries of the Eastern Mediterranean Region and describing their progress towards the global salt reduction target. A systematic review of published and grey literature was conducted. Key characteristics of strategies were extracted and classified according to a pre-defined framework: salt intake assessments; leadership and strategic approach; implementation strategies; monitoring and evaluation of program impact. Salt intake levels were estimated in 15 out of the 22 countries (68%), while national salt reduction initiatives were identified in 13 (59%). The majority of countries were found to implement multifaceted reduction interventions, characterized by a combination of two or more implementation strategies. The least common implementation strategy was taxation, while the most common was reformulation (100%), followed by consumer education (77%), initiatives in specific settings (54%), and front of pack labelling (46%). Monitoring activities were conducted by few countries (27%), while impact evaluations were lacking. Despite the ongoing salt reduction efforts in several countries of the region, more action is needed to initiate reduction programs in countries that are lagging behind, and to ensure rigorous implementation and evaluations of ongoing programs. Such efforts are vital for the achievement of the targeted 30% reduction in salt intake.

## 1. Introduction

Cardiovascular diseases (CVD) are the leading cause of mortality worldwide, resulting in around 17.9 million deaths each year and representing 31% of global deaths [1]. The principal risk factor for CVD is high blood pressure (HBP) [2], with excessive sodium intake being recognized as a significant contributor to both HBP and CVD mortality [3]. Based on the recent Global Burden of Disease (GBD) study, HBP was found to account for 10.4 million deaths and 218 million disability-adjusted life years (DALYs) in 2017, while excess sodium intake contributed to 3.2 million deaths and 70 million DALYs [4].

The World Health Organization (WHO) has recommended the reduction of salt/sodium intakes as a ‘best buy’, acknowledging it as one of the most cost-effective and feasible approaches to reduce the risk of CVD, stroke, and coronary heart disease (CHD) [5]. While several observational analyses have disputed the nature and magnitude of the association between sodium and vascular outcomes [6,7,8,9], available evidence supports the lowering of the population’s sodium consumption as an effective public health strategy [10,11]. A recent systematic review of prospective studies showed a direct association between urinary excretion of sodium and the risk of CVD mortality, this association being more significant at sodium intakes exceeding 2.4 g/day [11]. The United Kingdom’s sodium reduction strategy also provided support with regards to the feasibility and impact of such interventions, showing a 15% decrease in the population’s sodium intake between 2003 and 2011 [12], paralleled by a reduction in the population’s average blood pressure by 3/1.4 mm Hg [13]. In 2013, at the 66th World Health Assembly, the WHO Member States adopted the global target of a 30% relative reduction in mean population intake of sodium by the year 2025, against a 2010 baseline (Target 4) [14]. This target is one of the nine voluntary global targets for the worldwide reduction of non-communicable diseases (NCDs), including CVDs [15]. Efforts to reach the global target on sodium reduction are also expected to contribute towards achieving the Sustainable Development Goals (SDGs), including Target 3.4 of reducing premature NCD-related mortality by at least a third [16].

The Eastern Mediterranean Region (EMR), which includes 22 countries and a population of approximately 580 million people, is a region that harbors a high burden of CVD [17]. A recent study based on the GBD 2015 data for the EMR showed that CVD contributed to the loss of nearly 33 million years of life due to premature mortality or disability, and to more than 1.3 million deaths, thus accounting for approximately one-third of all deaths in the region [18]. This study also showed that HBP was the most important risk factor for CVD in the EMR [18], and that high sodium intakes were amongst the leading dietary risk factors [18]. Over the last decade, countries of the EMR have been working towards attaining the global NCD targets, including the reduction of mean population sodium intake by 30% [19]. In fact, the WHO Regional strategy on nutrition 2010–2019 [20] has included a number of specific objectives relating to the prevention of diet-related NCDs, with one of these objectives focusing specifically on the reduction of mean population intake of salt/sodium by 30% [20]. This was followed by the adoption of the new Strategy on Nutrition for the Eastern Mediterranean Region 2020–2030 in October 2019, to further assist countries of the region in fostering their action on nutrition, including the reduction of salt/sodium intakes [21]. However, up till today, there has been no systematic and in-depth appraisal that focuses specifically on national salt reduction initiatives in countries of the EMR. It is in this context that we conducted this systematic review with the aim of identifying and documenting existing national salt reduction strategies in countries of the EMR, providing an overview of initiatives that are implemented by countries of the region to reduce population salt intake, and describing progress towards the global NCD salt reduction target of 2025.

## 2. Materials and Methods

**Figure 1:**
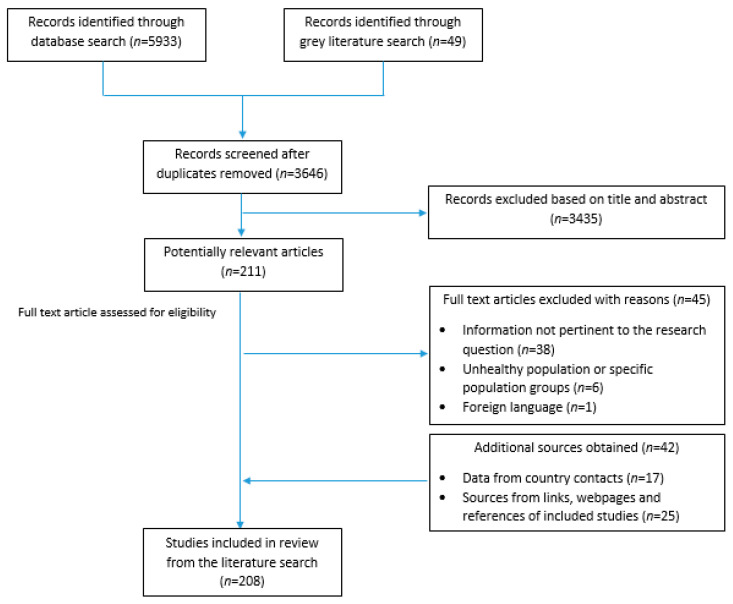
Search and identification process of potential references from the literature.

The methods and search strategy adopted in the present study were based on the approach described by Trieu et al. and Santos et al. [10,22]. In brief, data pertinent to population salt reduction initiatives were obtained through a series of steps allowing for maximum coverage of the 22 countries of the EMR. This included a search of peer-reviewed and grey literature published up to March 2021, as well as seeking Supplementary Information by directly contacting program country leaders (Figure 1).

### 2.1. Search Strategy

Eleven electronic databases were searched between 26 and 31 January 2021, including four Arabic databases. Accordingly, the following databases were consulted for the search: CAB Direct, Directory of Open Access Journals, Google Scholar, MEDLINE OVID interface (1946 to present), PubMed, Scopus, Web of Science Core Collections, Al Manhal, Arab World Research Source (AWRS), E-Marefa and Iraqi Academic Scientific Journals (IASJ); the last four being specific to the Arab region. In addition to using controlled vocabulary (MeSH in PubMed and MEDLINE), a comprehensive list of search terms was used in the title/abstract/keyword fields to cover the four concepts (1) salt, (2) reduction OR intake, (3) policy, and (4) EMR countries. The detailed list of search terms is shown in Appendix A, and an example of a database search is shown in Table S1. The search was limited to materials published post 1995 in English, Arabic, and French languages only. Newly published articles after the execution of the initial search were identified through email alerts (up until 22 March 2021).

A search of the grey literature was also conducted, using OpenGrey, Google, World Action on Salt and Health, the Global Database on the Implementation of Nutrition Action (GINA), the WHO EMRO (Regional Office for the Eastern Mediterranean) website and governmental websites (e.g., Ministries of Health). The search was limited to the English, Arabic, and French languages only and published post 1995.

Articles identified through the search of online databases and grey literature were exported to EndNote X9 (Version 18.0.0.10063). Two independent researchers (MT and AC) screened the titles, abstracts and full text articles of the potentially relevant articles, based on the inclusion and exclusion criteria described in the section below. The few discrepancies from the two screening stages were discussed and resolved by the two researchers.

### 2.2. Inclusion and Exclusion Criteria

Articles were included in the review if they provided information pertinent to salt intake assessment, or the development, implementation, monitoring, progress, or evaluation of national salt reduction initiatives at the national level. As per the approach adopted by Trieu et al. [10], the definition of a national salt reduction initiative was based on having the involvement of a governmental entity, and at least one of the following components: (1) a document that presents a national plan of action to decrease the population salt intake; (2) a program of work that involves the food industry for product reformulation, (3) consumers’ education programs or awareness campaigns aimed at improving knowledge, attitudes, and behavior (KAB) towards salt; (4) front of pack labelling (FOPL) schemes that specifically include salt, (5) taxation policies targeting high-salt foods, or unhealthy foods if the definition of the latter includes high salt or sodium; and (6) salt reduction initiatives in specific settings (schools, hospitals, workplaces).

Articles were excluded if they were based on randomized-control trials or case-control studies; focusing on unhealthy individuals or specific populations (pregnant women, individuals on therapeutic diets, etc.). Individual articles were included if they were published after 1995, or if they were in languages other than English, Arabic, and French.

### 2.3. Data Extraction

Data extraction was then conducted by one researcher (MT), and a second researcher (LN) reviewed the data for accuracy. Any inconsistency was resolved through discussion until reaching consensus. For each national salt reduction strategy, the key characteristics were entered into a database that was constructed by the researchers, and examined in relation to baseline assessments (population intake, levels in food products, KAB), leadership and strategic approach, implementation strategies (food reformulation, consumer education, FOPL, interventions in public institution settings, taxation), monitoring (population intake, levels in food products, KAB), and evaluation of program impact [10,22,23].

### 2.4. Seeking Supplementary Information

A standardized questionnaire was developed based on the one adopted by Trieu et al. [10] and sent to country experts or program leaders in the region to verify and obtain supplementary country-specific data. Experts or program leaders were invited to collaborate on the study and fill the questionnaire and/or pass it on to their contacts to gather more information and provide the needed details. The additional data obtained from the questionnaires were utilized to update the database.

### 2.5. Analysis

Core characteristics for each national salt reduction strategy were entered into a database according to a pre-defined framework that includes baseline assessments; strategic approach; types of implementation strategies; monitoring data; and evaluation of program impact. Countries were then classified as ‘having a developed strategy’, ‘having a planned strategy’, or ‘having no strategy’. Strategies were considered to be “planned” if the salt reduction initiatives were still being developed or if the strategic action plan had been already developed but without evidence of implementation [10]. A quantitative assessment of the proportion of countries reporting each characteristic was performed, based on percentages.

## 3. Results

### 3.1. Search Results

A total of 208 peer-reviewed articles, grey literature documents, and websites were obtained from the literature search; 166 were peer-reviewed articles and 42 were additional sources obtained from links, webpages, and references from within the included studies, or from country contacts via the completed questionnaires (Figure 1).

### 3.2. Salt Intake Assessment

In the EMR, seven countries (32%) including Afghanistan, Djibouti, Libya, Qatar, Somalia, Syria and Yemen do not have any estimate of salt intake in their population, (with the exception of estimates based on Bayesian modelling, which were not taken into consideration in this review) [24]. In contrast, 15 countries (68%) were found to have one or more estimates of population salt intake (Table S2), with some studies reporting salt intake at the national level while others targeted specific regions within countries [25,26,27,28,29,30,31,32,33,34,35,36,37,38,39,40,41,42,43,44,45,46,47,48,49,50,51,52,53,54,55,56,57,58,59,60,61,62,63,64,65,66,67,68,69,70,71,72,73,74,75,76,77,78,79,80,81,82,83,84,85,86,87,88]. Twenty-four urine collection, which is considered as the gold standard for the assessment of salt intake, was conducted in 11 countries of the region (50%) (Egypt, Iran, Iraq, Jordan, KSA, Lebanon, Morocco, Oman, Pakistan, Tunisia, and the UAE) (Table S2). Spot urinary excretion studies were also conducted in nine countries (41%) (Egypt, Iran, Jordan, Lebanon, Morocco, Oman, Pakistan, Palestine, and Sudan) (Table S2). Dietary assessment of salt intake based on the use of food frequency questionnaires (FFQs), 24 h recalls or records has also been conducted in 12 countries (55%), including Bahrain, Egypt, Iran, Jordan, KSA, Kuwait, Lebanon, Morocco, Oman, Pakistan, Tunisia, and the UAE. Household budget surveys have also been used in Jordan (Table S2).

**Figure 2:**
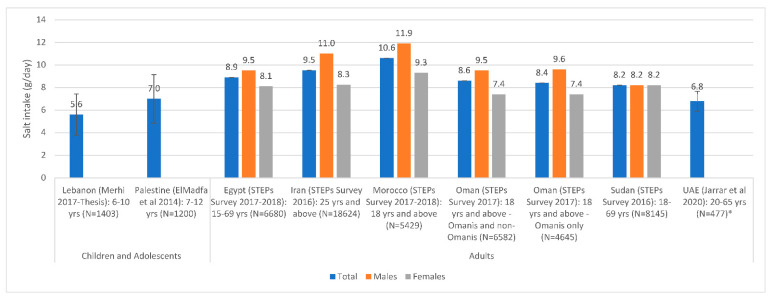
Salt intake estimates based on national urinary excretion studies in countries of the EMR#. Abbreviations: EMR: Eastern Mediterranean Region; UAE: United Arab Emirates. References: For Children and Adolescents: Lebanon: Merhi 2017–Thesis [63]; Palestine: ElMadfa et al., 2014 [81]. For Adults: Egypt: STEPs Survey 2017–2018 [29]; Iran: STEPs Survey 2016 [33,36]; Morocco: STEPs Survey 2017–2018 [89]; Oman: STEPs Survey 2017 [76,90]; Sudan: STEPs Survey 2016 [82]; UAE: Jarrar et al., 2020 [87]. *: All studies were based on spot urine assessment with the exception of UAE (Jarrar et al., 2020), which was based on a 24 h urinary assessment. #: Error bars (standard deviations around the mean) are presented, when available.

**Figure 3:**
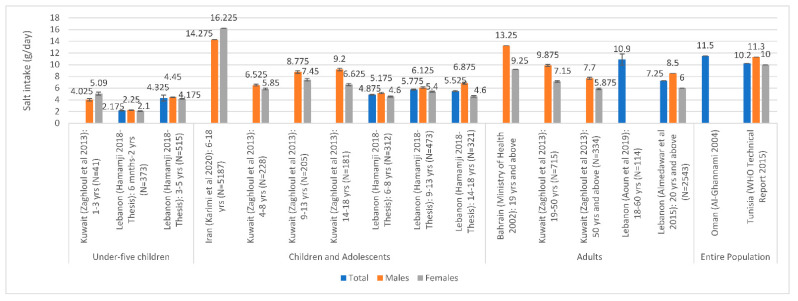
Salt intake estimates based on national dietary assessment studies conducted in countries of the EMR#. Abbreviations: EMR: Eastern Mediterranean Region; WHO: World Health Organization. References: For Underfive Children: Kuwait: Zaghloul et al., 2013 [59]; Lebanon: Hamamji 2018–Thesis [67]. For Children and Adolescents: Iran: Karimi et al., 2020 [45]; Kuwait: Zaghloul et al., 2013 [59]; Lebanon: Hamamji 2018–Thesis [67]. For Adults: Bahrain: Ministry of Health 2002 [25]; Kuwait: Zaghloul et al., 2013 [59]; Lebanon: Aoun et al., 2019 [68] and Almedawar et al., 2015 [69]. For the Entire Population: Oman: Al-Ghannami 2004 [77]; Tunisia: WHO Technical Report 2015 [84]. #: Error bars (standard deviations around the mean) are presented, when available.

The most recent national estimates of sodium/salt intake levels are presented in Figure 2 based on urinary excretion, and in Figure 3 based on dietary assessment. Based on urinary excretion, the highest level of salt intake was observed in Morocco (10.6 g/day), while the lowest was observed in Lebanon (5.6 g/day) and the UAE (6.8 g/day) (Figure 2). Based on dietary assessment, the highest levels of salt intake were observed amongst Iranian children and adolescents (14.3–16.2 g/day) and adults in Bahrain (9.3–13.3 g/day) and Lebanon (10.9 g/day). Per capita estimates were also high in Oman (11.5 g/day) and Tunisia (10.2 g/day) (Figure 3). Overall, Figure 2 and Figure 3 show that salt intake levels were all above the WHO upper limit of 5 g/day in all countries, except for data reported amongst under-5 children.

### 3.3. Assessment of Salt Levels in Food and Salt-Related KAB

Data regarding the baseline levels of salt/sodium in specific foods or meals were collected in 16 countries of the EMR (73%), including Bahrain, Egypt, Iran, Iraq, Jordan, KSA, Kuwait, Lebanon, Morocco, Oman, Pakistan, Palestine, Qatar, Tunisia, UAE, and Yemen [27,28,43,53,69,84,91,92,93,94,95,96,97,98,99,100,101,102,103,104,105,106,107,108,109,110,111,112,113,114,115,116,117,118,119,120,121,122,123,124,125]. These salt/sodium content data were collected based on food composition databases, food analysis, or industry self-reporting. Of the 22 EMR countries, the proportion measuring baseline KAB in relation to salt is estimated at 45%. These countries include Egypt, Iran, Jordan, KSA, Lebanon, Morocco, Oman, Pakistan, Sudan, and the UAE. Most of the KAB surveys were based on questions relating to knowledge of the adverse health effects of high salt intake, attitudes towards the reduction of salt intake and its importance, as well as consumers’ behavior such as adding salt to food prior to tasting, or cooking with low-salt products [29,30,52,54,70,75,79,82,87,88,125,126,127,128,129,130,131,132,133,134,135,136,137,138,139,140].

### 3.4. Countries with National Salt Reduction Strategies

As shown in Table 1, national salt reduction initiatives were identified in 13 out of the 22 countries of the EMR (59%). These 13 countries include Bahrain, Egypt, Iran, Jordan, KSA, Kuwait, Lebanon, Morocco, Oman, Palestine, Qatar, Tunisia, and the UAE. In their review, Lachat et al. (2013) mentioned that Sudan had a proposal for taxes on salty foods and that initiatives for promoting the reduction of salt intake in the population exist in Sudan and Djibouti [141]. However, we were not able to confirm or locate any further details related to these measures. Hence, for the purpose of this systematic review, Sudan and Djibouti were considered as “having a planned strategy” for the reduction of population salt intake. In addition, the GINA database included Iraq amongst countries that have implemented policies to reduce the population’s salt/sodium consumption [142]. More specifically, it referred to salt reduction in bread to a required percentage of 0.5% salt on a dry flour weight basis as of 2016 [143]. However, the GINA database has also indicated that this initiative was not adopted in the country [143]. Accordingly, and given that no other salt reduction initiatives were found during our systematic search, Iraq was not considered as having national salt reduction initiatives in this review.

### 3.5. Leadership and Strategic Approach

All of the identified strategies were mainly led by the government, except for Lebanon, where the salt reduction initiative was led by academia in collaboration with the government (Table 1). Except for Lebanon and Palestine, the remaining 11 countries (85%) have established targets for population salt intake. More specifically, Bahrain, Jordan, KSA, Kuwait, Oman, and Qatar have a target of 5 g of salt per day, as per the WHO recommendations. The target adopted by Egypt, Iran, Tunisia, and the UAE consists of a 30% relative reduction in salt intake, while that of Morocco is set at 10% reduction. As shown in Table 2, salt reduction strategies in countries of the region were part of broader initiatives targeting NCD or healthy diets and lifestyles.

### 3.6. Implementation Strategies

Except for Palestine, all the other 12 countries are implementing multifaceted salt reduction interventions, characterized by a combination of two or more implementation strategies (Table 1). The least common implementation strategy in countries of the EMR is taxation. Except for Qatar, where taxation of high salt products is planned, and for the UAE where taxation of unhealthy salty foods was included in the National Action Plan of 2017 (although not adopted yet) [185], none of the countries have initiatives that involve taxation. The most common salt reduction initiative in countries of the EMR is reformulation (all of the 13 countries, 100%), followed by consumer education (10/13 countries; 77%), initiatives in specific settings (7/13 countries; 54%), and FOPL (6/13 countries; 46%). Table 1 shows the details for the activities and initiatives implemented by the various EMR countries.

#### 3.6.1. Reformulation

Except for Lebanon, all the reformulation initiatives in the various countries were led by governmental authorities, while engaging with the food industry. The most common food product targeted by these reformulation initiatives is bread, and in eight countries of the region, reformulation initiatives are mandatory (Bahrain, Iran, Jordan, KSA, Kuwait, Oman, Palestine, and Qatar) (8/13 countries; 62%). Some countries have further expanded the reformulation to other food products such as cheese, salty snacks, canned foods, etc. (Iran, Jordan, KSA, Kuwait, and the UAE) (5/13 countries; 38%).

#### 3.6.2. Consumer Education

Enhancing consumer awareness and education in relation to dietary salt intake is a component of most strategies implemented in countries of the EMR. All except Bahrain, Kuwait, and Palestine have implemented consumer education campaigns (Table 1). The majority of these awareness campaigns were led by the government, except for Lebanon, where this component was led by academicians. The majority of these initiatives (80%) were specific to salt, while others were broader campaigns in relation to healthy diets that had also included a salt component (20%). In most countries, consumer awareness and education activities were implemented in conjunction with other salt reduction initiatives.

#### 3.6.3. Front of Pack Labelling

Six countries (27%) were found to have FOPL initiatives related to salt. These countries include Bahrain, Iran, KSA, Morocco, Tunisia, and the UAE. Mandatory traffic light labelling scheme is implemented in Iran for packaged foods. The traffic light labelling scheme is also implemented in KSA and the UAE on a voluntary basis, but it is planned to become mandatory as of 2022 in the UAE [167]. Mandatory FOPL has been also adopted by Morocco, based on the Nutriscore labelling scheme. Bahrain is implementing mandatory labelling of baked products (traditional and others), requiring the specification of the amount of added salt, while in Tunisia, mandatory FOPL of salt content is planned, but not adopted yet (Table 1).

#### 3.6.4. Interventions in Specific Settings

Five countries (23%) are implementing salt-reduction initiatives in specific settings. These include Egypt, KSA, Kuwait, Qatar, and the UAE. More specifically, health education initiatives that incorporate a salt reduction component are implemented in Egypt under the 100 Million Health initiative, in KSA under the Healthy Food Strategy, and in Qatar under the Qatar Dietary Guidelines, the Food and Beverage Guidelines and the School Canteen Guidelines, targeting healthcare facilities, schools, workplaces, and the community in general. KSA is also implementing a voluntary procurement policy to limit the procurement and hence the availability of high salt foods in these settings. Kuwait is implementing hospital menu modification as well as the labelling of foods sold in governmental hospitals, cafeterias, and canteens to reduce the consumption of high salt foods. In the UAE, the initiative related to healthy recipes for the school lunch bag promotes the consumption of low salt food. Other planned initiatives that have not been adopted yet include the creation of supportive environments by offering low salt foods in public places, schools, and the workplace in Tunisia and the limiting of the availability of high salt foods in schools in Oman.

### 3.7. Monitoring

Multisectoral national committees have already been established in Egypt, Iran, Jordan, Kuwait, Oman, Qatar, Tunisia, and the UAE (36%) to strategize and monitor the implementation of national salt reduction strategies [144,148,185].

In Kuwait, where 80% of total bread production is produced locally by Kuwait Flour Mills and Bakeries Company (KFMBC), monitoring activities showed that the actual percentage of salt reduction in bread ranged from 12–16%, mainly due to technical difficulties related to the taste and texture of the products [61]. In addition, as part of the strategy to promote and support nutrition in school children (6–18 years old) in Kuwait, whereby corn and potato crisp products had mandatory salt content targets, the results of monitoring activities showed that 65% of crisps products still exceeded the 1.5 g/100 g target [61].

In Bahrain, a ministerial decree was formulated in 2014 in order to establish a multisectoral national committee that will work on the reduction of salt in bread products [92]. More specifically, the work of this committee will focus on developing an action plan for salt reduction, enforcing food labelling to include salt content, and developing legislation for the monitoring of salt reduction implementation [92]. Although a plan for the sampling of bread to assess for salt compliance has been developed [145,189], results pertinent to this monitoring were not published. In KSA, the Saudi Food and Drug Authority (SFDA) has conducted, in 2019, several monitoring activities, the main approach being the collection of food samples from the market to check the extent of compliance with the salt regulation set by SFDA [120]. Accordingly, compliance to the sodium/salt reduction strategies was estimated at 47% (123 items out of 261), the highest compliance having been reported for pasta (100%), butter (88%), cooking sauces (92%), and pizza (80%). The lowest compliance was observed in ready-made meals (0%), cereals/canned vegetables/legumes (20%), canned vegetables (12%), and cheeses (22%). In Qatar, the Ministry of Public Health (MOPH) is monitoring the implementation of the salt reduction policy in bread in order to ensure its sustainable implementation [148]. More specifically, bread samples are regularly collected and sent to the MOPH Central Food Laboratory to determine salt content and identify deviations from the target of 0.8% salt levels (unpublished). Accordingly, the nutritional content of any new food and beverage product that is to be potentially supplied within the schools’ canteen is inspected prior to its inclusion in the canteen. In the UAE, the monitoring and evaluation of the impact of the salt reduction is included in the 2017 National Action Plan, but data on monitoring and evaluation were not found [185].

In Morocco, the knowledge of bakers as well as their commitment towards the national salt strategy was assessed [125,192]. A total of 432 bakeries from all regions in Morocco were recruited in 2018 [125] and questionnaires were administered to the bakers. The study findings showed that around 73% of the interviewed bakers were not aware of the recommendations related to the progressive reduction of salt content in bread, and 60.32% did not respect the national recommendations of 10 g of salt/kg of flour, while 89.6% expressed their interest in getting committed to the process in the next two years [125]. Another study conducted in the region of Casablanca (Morocco) gave similar results related to the bakers’ knowledge [192]. In addition, bread samples were collected from 160 bakeries in Casablanca [192]. The results showed that for white bread, average salt levels decreased from 17.8 g/kg in 2011 to 13.1 g/kg in 2016 (more than 25% reduction), while highlighting large discrepancies between different bakeries, with a range of 10.5 to 19.6 g/kg [192]. In Tunisia, and more specifically, in Bizerte city, where the pilot phase of the national action plan is implemented, bread samples were collected randomly from bakeries once per week during the first three months and then, once every three months during the next period of the protocol, which extended between 2015 and 2018 [182]. Salt levels in bread was estimated at 1.7 g/100 g in 2015, and this level reached 1.1 g/100 g in 2018, i.e., after 3 years of implementing the salt reduction program. Hence, the average salt reduction was of 35% in bread [182]. In parallel, a pilot study on consumer acceptability of the salt-reduced bread was conducted. Results on the perception of bread saltiness by Tunisian consumers showed that a 30% salt reduction in the bread was not detected by the majority of the consumers, with 79% of the participants reporting that the salt-reduced bread had normal saltiness [182].

### 3.8. Impact Assessment

Studies or reports on impact assessment were not found for any country of the EMR.

## 4. Discussion

This systematic review is the first to focus on salt reduction initiatives in countries of the EMR, a region that harbors a high burden of cardiovascular morbidity and mortality. It showed that, out of 22 countries in the EMR, 15 had one or more estimates of salt intake in the population (68%), and 13 had national salt reduction strategies (59%). The most common intervention was food reformulation through engagement with the food industry, while the least common was taxation.

Based on the most recent national studies conducted in countries of the region, the population’s salt intake levels remain high, exceeding the WHO upper limit of 5 g/day in all countries of the EMR. In most countries, these estimates are based on studies or surveys that were collected prior to the implementation of national salt reduction initiatives and thus serve as baseline values of population salt intake. Interestingly, many of these studies have also reported on the major dietary contributors to salt intake, an information that ought to be considered in policy development and implementation. For instance, Egypt, Iraq, KSA, Kuwait, and Lebanon have identified bread and other bakery products, processed foods, ready meals, processed meats, as well as cheeses and dairy products, as the main food sources amongst adults [27,28,29,51,58,61,62,69]. In Iran, bread, cheese, and snacks were identified as the major contributors to salt intake amongst children aged 3–10 years [42,43], while studies conducted in Morocco reported that cereals and cereal-based products, followed by spices and condiments, dairy products, and meat products were the main sources amongst adolescents [70,74].

The number of countries implementing salt reduction initiatives in the EMR has grown from 2 in 2014, as reported by Trieu et al. (2015) [10] in their global review, to 13 in 2021. This progress has been described by Santos et al. (2021) as the largest increase worldwide, reflecting the concerted efforts led by the WHO EMRO [22], which has spearheaded, over the past decade, the development of policy guidance for Member States to reduce population salt intake; set up a regional monitoring mechanism; developed a regional protocol on 24 h urinary sodium; and supported a network of regional research institution to conduct 24 h urinary sodium excretion measurements and assess dietary sodium intake [148]. The nutrition strategy 2020–2030 developed by the WHO EMRO has also included specific objectives related to the reduction of the mean population’s intake of salt/sodium, in addition to recommending a number of priority actions for Member States [21].

Unlike findings reported by Santos et al. (2021), whereby interventions in settings were the most common type of salt reduction initiatives at the global level, the most common salt reduction approach in the EMR was food reformulation, which was adopted by 13 countries in the region [22]. Food reformulation was followed by consumer education interventions (10 countries), initiatives in specific settings (7 countries), and FOPL schemes (6 countries), while taxation measures have not been implemented yet. At the global level, taxation also appears as the least implemented salt reduction intervention with only 5% of countries worldwide adopting this measure [22].

Food reformulation interventions in the region have mostly focused on bread, the staple food in most countries of the EMR [91]. Studies conducted in the region have in fact shown that bread was amongst the largest contributor to salt/sodium intake in the diet [28,84,91,109]. Few countries such as Iran, Jordan, KSA, Kuwait, and the UAE have further expanded their food reformulation interventions to other food products, including dairy, salty snacks, processed meat, etc. This is encouraging given that studies conducted in EMR countries have identified important contributors to salt, other than bread, including cereals and cereal products [103], dairy [103,193], prepared meals [58], salted foods [193], and processed meats [193].

Consumer education was the second-most common intervention in countries of the region, with the aim of raising awareness about salt, its main dietary sources, and its health effects. Knowledge studies conducted in various countries of the EMR had in fact shown that consumers have poor knowledge related to the potential adverse effects of excessive salt consumption on health [79,126,128,130], have a poor understanding of salt-related information on the food label [88,130], and are not able to identify the main sources of salt in the diet [88,130]. The available few studies on salt-related attitude in the region have also revealed that only a small proportion of consumers were concerned about the amount of salt consumed in their diets [88]. At the same time, suboptimal practices were identified, with a small percentage of consumers reporting to check salt-related information on the food label [88,130], while the vast majority reported to add salt during cooking or at the table [52,79,88,126,127,131,133,134,140]. It is recommended that countries of the region consult these KAB findings when developing or further tailoring their consumer education initiatives in order to address culture-specific gaps in knowledge and attitude towards salt reduction. In addition, acknowledging that FOPL can be an important element of national strategies aimed at improving the population’s diets, a positive finding of this review is the fact that FOPL is being implemented or planned in six countries of the region [167]. Acknowledging that data on the main dietary contributors to salt intake is available in several countries of the region, this data ought to be consulted in a holistic manner prior to the development and implementation of salt reduction strategies to ensure that these strategies are better tailored to the local context.

This review showed that 13 countries of the EMR are implementing multifaceted national salt reduction initiatives, which is an encouraging observation, given the potential of such strategies in influencing salt intake level and hence NCD risk [194]. In fact, salt reduction is increasingly recognized as a ‘best buy’ intervention, which is not only cost-effective, but also affordable, feasible, and culturally acceptable in any context resource setting [5]. In the EMR, the potential impact of three policies to reduce dietary salt intake were assessed: a health promotion campaign, the labelling of packaged food, and the mandatory reformulation of salt content in processed food [195]. Cumulative population health effects were quantified as life years gained (LYG) over a 10-year time frame, and all costs were calculated using the 2010 purchasing power parity exchange rates. The results showed that for Palestine, the combination of all three policies, which would lead to a 30% reduction in salt intake, would result in an estimated cost savings of $6,000,000 and 2682 life-years gains. In Syria, the combination of these policies would lead to estimated cost savings of $39,000,000 and 31,674 life-years gained [195], while in Tunisia, this would result in estimated cost savings of $235,000,000 and 6455 life-years gained [195]. Another study conducted in Syria showed that the combination of reformulation with labelling, or the comprehensive policy that includes all three approaches (health promotion campaigns; food reformulation and food labelling) were the most promising salt reduction strategies to reduce CHD mortality in Syria. Approximately 32,000 life years would be saved in Syria, over a10-year period, if all three interventions were implemented [196].

This review showed that some countries of the region have incorporated a legislative component within their strategies instead of implementing solely voluntary initiatives. In fact, a mandatory approach was adopted in almost half of the countries implementing food reformulation initiatives (8 out of the 13). These countries include Bahrain, Iran, Jordan, KSA, Kuwait, Oman, Palestine, and Qatar. In addition, all of the six countries that are already implementing or planning to implement FOPL have incorporated a legislative component (Bahrain, Iran, KSA, Morocco, Tunisia, and the UAE). Kuwait has also adopted a mandatory approach within its food procurement policies, specifying salt standards for foods in public institution settings such as public schools and hospitals. Previous modeling studies as well as systematic reviews have shown that mandatory or legislative approaches tend to be more effective, producing larger reductions in salt intake levels within the population [197,198,199].

The implementation of clear monitoring approaches is crucial to demonstrate program effectiveness, and to incite greater changes, especially for voluntary strategies [200]. In the EMR, six countries only (Jordan, KSA, Morocco, Oman, Qatar and Tunisia) have established mechanisms for the monitoring of sodium content in one or more food categories, and using laboratory analysis. The latter is undoubtedly a highly accurate method for salt level determination, but, given its cost, it may cover only limited range of products rather than the whole food supply [10]. There may be a need for comprehensive surveys of salt levels in food products, based on validated product label data, an approach that can complement laboratory analysis of specific foods, to ensure that progress is made over a larger scale [34].

The assessment of the impact of salt reduction programs on population salt intake levels was not performed in any country of the EMR. For the majority of the countries, there were no available studies or surveys that have assessed salt intake after the implementation of national salt reduction initiatives. This is likely due to the fact that most salt reduction initiatives in the region are relatively recent and there has been insufficient time to assess impact. For two countries, Kuwait and Morocco, we were able to identify published studies that were conducted before and after the national initiative implementation. Compared to a baseline estimate of 4 g of sodium per day amongst adults in 2008 in Kuwait [60], the national survey of 2014 reported an intake ranging between 4 and 5.4 g of sodium per day [61,62], i.e., one year after the launch of salt reduction in bread. In both studies, the assessment of sodium intake was based on questionnaires, but details about these questionnaires and whether they were comparable were not found. In Morocco, an assessment of salt intake was conducted in 2014, in the central regions of the country via 24-h urinary excretion, reporting an estimate of 2.8 g of sodium per day in adults [72,73]. The national 2017–2018 survey, which was based on the collection of spot urine samples, reported an intake of 4.24 g of sodium per day amongst adults [70]. The 2017–2018 survey was conducted approximately two years after the initiation of salt reduction initiatives. Thus, although data may not be directly comparable, given the methodological differences between the studies, the available data does not suggest a reduction in the population’s salt intake. There is a crucial need for comparative surveys to measure change in salt intake over time [38,39] and hence document the programs’ impact in countries of the EMR. Despite being considered the gold standard for salt intake assessment, the 24 h urinary sodium excretion approach can be costly while also imposing a high burden on participants, which limits its applicability to many countries of the region [20]. The collection of spot urine samples to determine 24 h urinary sodium excretion may thus be more applicable to large population surveys [201] and the WHO has in fact produced guidance for the monitoring of salt intake as part of STEPs, which can be adopted by countries of the region [20]. Many countries of the EMR have in fact recently adopted the STEPs spot urine protocol for salt intake assessment, but these estimates are available for one point in time only, which cannot document progress. Standardized comparable dietary approaches to measuring salt intake may also be used to document change in salt intake over time. Although dietary approaches are likely to underestimate salt intake, if the adopted method of assessment is consistent, it can still be a useful measure of change over time [201,202]. Since the regular measurement of changes in population salt intake may be complex and costly, the incorporation of process evaluations that examine the strategy implementation and its progress, collects process indicators, and identifies existing barriers and facilitators of implementation are probably more feasible and informative for identifying specific areas for improvement [203].

Government-led monitoring activities of salt-related KAB to assess the impact of consumer education campaigns were not found. In Morocco, the bakers’ knowledge in relation to salt reduction in bread was assessed [125], but progress could not be assessed given the lack of baseline estimates. We were able to identify published studies that have assessed salt-related KAB before the implementation of consumer awareness initiatives and afterwards in four countries of the region: Jordan, KSA, Oman, and the UAE [52,54,87,88,131,133,134,137,139,204]. However, the fact that different population groups and different questionnaires were used to assess KAB at the two distinct time points within the countries would undoubtedly limit the comparability of the data. It is crucial that more regular evaluations of national salt reduction initiatives be conducted in countries of the region, not only to document impact at the end of the intervention, but also interim evaluations during the life of the strategy, which can allow for the incorporation of essential adaptations to foster the strategy’s effectiveness [203,205].

This review has a number of strengths and limitations. This is the first systematic review of exiting salt reduction initiatives in countries of the EMR, their implementation, and progress towards achieving the new global target for salt reduction. In addition to the systematic search of databases and grey literature, questionnaires were sent to countries identified as having existing salt reduction initiatives and/or a country salt reduction contact person to verify and obtain supplementary country-specific data. Although not all country contacts were identified and we had some non-respondents, the triangulation of data from multiple sources allowed us to document the implementation of strategies, and present the information in a standardized manner. Through this, it is unlikely that any major salt reduction initiatives were missed, although this possibility cannot be totally excluded. Whilst one of the main strengths of the review is the fact that it included a comprehensive search of the grey literature, encompassing government reports, presentations or questionnaires completed by country program officers, a potential limitation arising from this approach is that the methodological rigor within some of the reports is unknown. More specifically, the robustness of the studies and the quality of the data used for the assessment of salt intake, salt levels in foods, and consumer KAB were not evaluated and hence should be interpreted with caution. It is also important to note that salt intake estimation may be limited by low participation and response rates, particularly in 24 h urine collection, which may result in sampling bias; hence, intake estimates ought to be interpreted with caution [206]. Finally, this paper did not review data pertinent to CVD incidence, CVD mortality, or prevalence of high blood pressure in countries of the region, which may have provided a deeper insight as to the impact of the study findings.

## 5. Conclusions

In conclusion, this study showed that 15 out of 22 countries of the EMR had one or more estimates of salt intake in the population (68%), and that 13 had implemented national salt reduction strategies (59%). The most common intervention was food reformulation, followed by consumer education interventions, initiatives in specific settings, and fourthly FOPL schemes. The impact of these interventions on the population salt intake level was not assessed, in any country of the EMR. However, based on the most recent national salt intake data, the majority of which were obtained prior to the implementation of salt reduction strategies, salt intake remains high in countries of the EMR, exceeding the WHO upper limit of 5 g/day. Despite the ongoing salt reduction efforts in several countries of the EMR, more action is needed to make sure that countries improve their monitoring activities, evaluate their strategies, and accelerate their efforts to meet the targeted 30% in salt intake in 2025. Countries of the region ought to develop well-designed monitoring activities to document progress within each intervention, identify gaps, and understand how the implementation process could be enhanced for better results and effectiveness.

## Figures and Tables

**Table 1:** Salt reduction implementation strategies in countries of the EMR.

Country	Engagement with Industries and Reformulation	Consumer Education	Front of Pack Labelling	Work in Specific Settings
Bahrain	Name of initiative: Determining the percentage of table salt to be added to bakery products Year: 2018 Leadership: Led by the government (MOH) Approach: Salt reduction in Arabic breads: Mandatory (supported by ministerial decree): A 20% annual reduction of salt level in traditional Arab breads from their current levels for five years until reaching the required percentage of 0.5% salt on a dry flour weight basis [144] Mandatory: Reduction in the amount of added salt in baked products during three years starting in 2018 and reaching a percentage of 0.5% salt [145,146] Cheese is under study [144]		Name of initiative: Determining the percentage of table salt to be added to bakery products Year: 2018 Leadership: Led by the government (MOH) Approach: Mandatory: Nutrition labels of baked products (traditional and European) should specify the amount of added salt [146]	
Egypt	Name of initiative: NA Year: 2017 Leadership: Led by government (MOHAP) Approach: Mandatory: ministerial decree to reduce by 30% the salt content of subsidized Baladi Bread—Planned target [31] Other initiatives: Voluntary: Training of bakeries on 20% reduction of salt in bread (Information provided by NFP)	Name of initiative: Pilot Implementation of 20% Salt Reduction in Subsidized Baladi Bread in Great Cairo Year: 2018–2019 Leadership: Led by the government Approach: Awareness campaigns (Information provided by NFP) Name of initiative: Brief Health Education sessions Year: NA Leadership: NA Approach: Awareness activities to increase consumers’ awareness towards hazards of high salt consumption and motivate them to reduce salt consumption (Information provided by NFP)		Name of initiative: 100 Million Health Initiative Year: 2019 Leadership: Led by the government (MOH) Approach: Education Setting: Hospital, school, workplace (Information provided by NFP)
Iran	Name of initiative: Adopted legislation for salt levels in several products Leadership: Led by the government Approach: Mandatory 2015: Establishing maximum salt levels in most commonly consumed canned foods such as tomato paste and salty snacks, and all types of bread (1.8%) [91,147,148] 2016–2017: The standard of salt in bread was further decreased to 1% [149,150,151,152]. 2015: The standard of salt in cheese was decreased from 4% to 3% [152,153] 2015: The standard of salt in dough (fermented drink) was decreased from 1% to 0.8% [152,154] 2018: Salt use has been banned in probiotic yogurts [152,155] Improvement of nutrition in public places through policies for reduced salt in food industries and restaurants; sensitizing food producers regarding the reduction of salt in food products [141] (No further details found)	Public education through TV regarding low salt consumption [141] (No further details found)	Name of initiative: Nutritional traffic light labelling Year: 2016 Leadership: Led by the government Approach: Mandatory for all imported and domestic packaged foods, except for products that are not chemically processed or formulated, such as vegetables, spices, vinegar, lemon juice, tea, infusions, coffee, honey, dates, flour, and barberry [152]	
Jordan	Name of initiative: Reduction of salt in Arabic bread Year: 2019 Leadership: Led by the government (part of reducing salt, sugar, trans fat consumption) Approach: Mandatory for bread; based on partnership with bakeries Target for breads: Less than 1% of dry weight [156] (planned) Name of initiative: NA Year: NA Leadership: Led by the government Approach: Revised already existing legislation to develop benchmarks for salt content in highly consumed foods, including cheeses [144,148] (No further details found)	Name of initiative: Low salt, sugar, and trans-fat consumption Year: 2018 Leadership: Led by the government (MOH) Approach: Social marketing (e.g., awareness campaigns), dietary guidelines health promotion campaign (No further details found) *Information provided by NFP* Develop national recommendations for the reduction of salt intake [141] (No further details found)		
KSA	Name of initiative: Healthy Food Strategy, vision 2030 Year: 2018 Leadership: Led by the government (SFDA) Approach: Voluntary: SFDA.FD 59 “salt limits in food product” Mandatory: SFDA.FD 2362 “General requirements for bread production” Mandatory: SFDA.FD 57 “Yogurt, Flavoured Yogurt, and Yogurt Drink” Food categories reformulated: Breads, processed meats, ready meals, breakfast cereals, cheeses, butter and margarines, salty snacks, biscuits and cakes, and soups and sauces Targets for breads (mandatory): 1.0 g/100 g of bread (all types of bread) [157,158,159,160] Mandatory salt limit for yogurt drink (Ayran Laban): 1 g/100 mL [160]	Name of initiative: Healthy Food Strategy, vision 2030 Year: 2018 Leadership: Led by the government (SFDA) Approach: Social marketing, events, social media posts [159,161] Name of initiative: Warns of Salt Excessive Consumption, Presents Substitutes Year: 2017–2020 Leadership: Led by the government (MOH and SFDA) Approach: Awareness video, guides and campaigns [162,163,164,165]	Name of initiative: Healthy Food Strategy, vision 2030 [159] Year: 2018 Leadership: Led by the government (SFDA) Approach: Voluntary for all food categories SFDA.FD 42 “Traffic light labeling” [166,167] - Low (green): Less than or equal to 0.3 g per 100 g - Medium (Orange): Greater than 0.3 g to less than or equal to 1.5 g per 100 g - High (Red): Greater than 1.5 g per 100 g For liquid food or beverages (per 100 mL): - Low (green): Less than or equal to 0.3 g per 100 mL - Medium (Orange): Greater than 0.3 g to less than or equal to 0.75 g per 100 mL - High (Red): Greater than 0.75 g per 100 mL	Name of initiative: Healthy Food Strategy, vision 2030 [159] Promoting healthy life style in work environment [168] Year: 2018 Leadership: Led by the government (SFDA) Approach: Voluntary; procurement policy and education Setting: Workplace, schools, and hospitals [121]
Kuwait	Name of initiative: Kuwaitis lower blood pressure by reducing salt in bread [62] Year: 2013 Leadership: Led by the government (MOH), in partnership with Kuwait Flour Mills and Bakeries Co (KFMB) Approach: Voluntary—implementation of a 10% reduction of salt in pita bread, which was achieved in March 2013, followed by another 10% reduction 6 months later in August 2013. The reduction included other white and whole wheat bread varieties [61] Revision of salt standard for cheese [144,148] Name of initiative: Strategy to promote and support nutrition in school children (6–18 years old) Year: 2017 Leadership: Led by the government—Community Nutrition Promotion Sector (CNPS) of the Public Authority for Food and Nutrition (PAFN) Approach: Mandatory: 6 corn and potato crisp companies were targeted Target of salt content ≤ 1.5 g/100 g [61]			Name of initiative: The role of Administration of Food and Nutrition MOH in controlling and prevention of non-communicable diseases in Kuwait Year: 2021 Leadership: Led by the government (Food and Nutrition Administration (FNA) on behalf of the Kuwait MOH)), in cooperation with Patient Helping Fund Society (PHFS) Approach: Mandatory- Apply the traffic light system on food items sold in governmental hospital cafeterias and canteens). Revision of hospital menus to decrease the levels of salt [169]
Lebanon	Name of initiative: Lebanese Action on Sodium and Health (LASH) Year: 2012 Leadership: Led by academia in collaboration with the government Approach: Voluntary—assesses intake and sources of salt; assesses baseline salt knowledge and improved knowledge; collaborating with the Ministry of Industry and MOH to develop national salt standards in bread [144,170]. Legislation on salt reduction should have been proposed to concerned members of Parliament) [171]	Name of initiative: Lebanese Action on Sodium and Health (LASH) Year: 2012 Leadership: Led by academia in collaboration with the government Approach: Aims to partner with a communications company to develop and launch a national salt reduction campaign in order to raise awareness on salt hazards and empower consumers to make better dietary choices [170]		
Morocco	Name of initiative: Reduction of salt content in food products Year: 2015 Leadership: Led by the government (MOH) [172] Voluntary for bread (No further details were identified)	Name of initiative: Salt awareness campaign for bakers Year: 2014 Leadership: Led by the government Approach: Awareness campaign for bakers (around 300 bakeries) in Grand Casablanca region; 70% committed to implement reduction [144,147,148] Name of initiative: Reduction of salt intake via campaigns Year: 2015 Leadership: Led by the government (MOH) Approach: Awareness campaign [172]	Name of initiative: Moroccan Nutrition Program and action plan 2017–2021 Year: NA Leadership: Led by the government Approach: Mandatory—FOP Nutrition logos, Nutri-Score (planned) [167]	
Oman	Name of initiative: Omani Standard for Bread [173] Year: 2019 Leadership: Led by the government (MOCI) Approach: The reduction of 10% of salt content started, as voluntary, in late 2015 and it was followed by another 10% to a total of 20% in the main three bakeries supplying most of the bread in the country [174,175] It became mandatory as of May 2019: 0.5% (0.5 g of salt in 100 g of bread) for flat bread (Arabic bread) [175] For other kinds of bread such as sliced bread or French bread: 1% (1 g of salt in 100 g of bread) [175] 30% reduction of salt in food products (planned) [144,175,176]	Name of initiative: Campaign for reduction of Salt consumption Year: Planned for 2021–2022 Leadership: Led by the government and NGOs Approach: Social marketing campaigns, TV advertising, and events (planned) (Information provided by NFP) Name of initiative: World Salt Awareness Week Year: 2018 Leadership: Led by the government (MOH, Nutrition department) Approach: In line with the world’s celebration of the World Salt Awareness Week, awareness activities are organized during the 12th to the 18th of March [177]		Name of initiative: NA Year: 2016 Leadership: Led by the government (MOH) Approach: Limit the availability of high salt items Setting: Schools (not adopted yet) [178]
Palestine	Name of initiative: National Health Strategy, 2021–2023 Year: 2019 Leadership: Led by the government (MOH) Approach: Food categories reformulated: gradual reduction of salt content in bread—mandatory; Targets set by year: 2019—0.9 g/100 g, 2021—0.8 g/100 g, 2022—0.7 g/100 g, 2023—0.6 g/100 g [179]			
Qatar	Name of initiative: WHO Salt Reduction in Bread Initiative Year: 2013 Leadership: Led by the government (MOPH) (part of Nutrition & Physical Activity Action Plan 2011–2016) [180] Approach: A 20% salt reduction in bread has already been initiated in the main national bakery (Mesaieed Bakery or Qbake). This reduction has also been achieved in other main bakeries of the country [92,144,148,181]. The aim is to reduce salt content in bread samples that contain more than 0.8% of salt [148] Targets set for bread are mandatory Name of initiative: Initiative to reduce fat, sugar, and salt consumption in Qatar Year: 2019 Leadership: Led by the government (MOPH) Approach: Approach done through meetings with the food industry to set voluntary commitments to fat, salt, and sugar reduction. Targets for salt levels in foods, and (Information provided by NFP)	Name of initiative: Food and Beverage Labelling and Calories Count Initiative at Restaurants and Coffee Shops Year: 2018 Leadership: Led by the government (MOPH and MOCI) Approach: Social marketing (e.g., campaigns, meetings with food industries, press releases, events (Information provided by NFP) Name of initiative: Salt Awareness Week (annually) Leadership: Led by the government (MOPH) Approach: Social marketing (e.g., campaigns, TV advertising (conducted TV and radio interviews), events (Information provided by NFP) Name of initiative: Initiative to reduce fat, sugar and salt consumption in Qatar Year: 2019 Leadership: Led by the government (MOPH) Approach: Social marketing (e.g., campaigns, TV advertising (conducted TV and radio interviews), events (Information provided by NFP)		Name of initiative: Food & Beverage Guidelines; School Canteen Guidelines for Cafeterias and Vending Machines; Educational sessions in schools and workplaces Year: Ongoing Leadership: Led by the government (MOPH) Approach: Education, guidelines Setting: School, hospital, workplace, and public places such as grocery stores and shopping malls (Information provided by NFP)
Tunisia	Name of initiative: Salt reduction program Year: 2015 Leadership: Led by the government Approach: Salt reduction program has already started in Bizerte city with the 22 bakeries; the program aims to progressively reduce the salt content of bread by 30%, voluntarily [144,147,182]. This is considered a pilot phase of the national action plan Name of initiative: Proposed reformulation of food products to contain less salt Year: 2018 Leadership: Led by the government (MOH) [86] (planned)	Name of initiative: Reducing salt intake and helping consumers make healthier choices that include less salt intakes Year: 2012 Leadership: Led by the government (MOH) Approach: NA [183] Name of initiative: Media campaigns to promote change in the intake of salt Year: 2018 Leadership: Led by the government (MOH) Approach: Not adopted [86]	Name of initiative: NA Year: 2018 Leadership: Led by the government (MOH) Approach: Mandatory—Recommend having salt content on the FOP (planned) [86,167,184].	Name of initiative: NA Year: 2018 Leadership: Led by the government (MOH) Approach: Create supportive environments in public places by offering low-salt options Setting: Public places, schools, workplaces (not adopted yet) [86]
UAE	Name of initiative: Reformulate food products (National Action Plan) Year: 2017 Leadership: Led by the government (MOHAP) Approach: Voluntary—reformulate breads to have less than 0.5% salt content and reduce salt levels in other food products such as pickles, cheeses, fast foods, snacks and other processed food (National Action Plan) (MOHAP Task Force on Reduction of Salt already established) [185]	Name of the initiative: Reduction of salt intake by 30% (National Action Plan) Year: 2017 Leadership: Led by the government (MOHAP) Approach: Recommendations for media campaigns and programs to spread awareness in the population (including nutrition labelling) and food industries, catering businesses including restaurants cafes, kiosk etc. (MOHAP Task Force on Reduction of Salt already established) [185,186] Name of initiative: Exclude salt from your food menu Year: 2019 Leadership: Led by the government (MOHAP) Approach: Instagram posts about high, medium and low salt foods [187] Name of initiative: Pay attention to the dangers of excessive salt intake! Year: 2018 Leadership: Led by the government (MOHAP) Approach: Instagram posts about food preparation tips to decrease salt [188]	Name of initiative: NA Year: 2020 Leadership: Led by the government Approach: Voluntary—traffic light labelling (Planned)—to become mandatory as of 2022 [167]	Name of initiative: Healthy recipes for school lunch bag Year: 2019 Leadership: Led by government Approach: Voluntary Setting: Schools [123]

Abbreviations: EMR: Eastern Mediterranean Region; FOP: front of pack; KSA: Kingdom of Saudi Arabia; MOCI: Ministry of Commerce and Industry; MOH: Ministry of Health; MOHAP: Ministry of Health and Prevention; MOPH: Ministry of Public Health; NA: not available; NFP: nutrition focal point; NGO: non-governmental organization; SFDA: Saudi Food and Drug Authority; UAE: United Arab Emirates; WHO: World Health Organization.

**Table 2:** National salt reduction strategies or action plans identified in countries of the EMR.

Country	National Strategy and/or Action Plan
Bahrain	Reduce intake of salt-containing foods to less than 5 g/day amongst adults (>18 years)—2012 (MOH) (National Action Plan for 15 years, for the Reduction of NCDs) [189]
Egypt	Reach a target of 20% relative reduction by 2021 and 10% relative reduction (9 g/day) by 2025 for adults—2017 (MOHP) (National Multisectoral Action Plan for Prevention and Control of NCDs 2017–2021) [31]
Iran	A 30% relative reduction in mean population intake of salt/sodium—2015 (Iranian National Committee for NCDs Prevention and Control) [190]
Jordan	Reduction of salt intake in the population to <5 g/day—2015 (MOH) (National Strategy And Plan Of Action Against Diabetes, Hypertension, Dyslipidemia And Obesity in Jordan) [191]
KSA	Salt reduction as per the WHO recommendation (no more than 5 g/day or 2000 mg of sodium/day)—2018 (SFDA) (Healthy Food Strategy, vision 2030) [157,159]
Kuwait	WHO recommended maximum of 5 g/day per adult—2013 (MOH) (Kuwaitis lower blood pressure by reducing salt in bread) [62]
Morocco	A reduction in population salt intake by 10% by 2025—2015 (MOH) (Prevention of NCDs: Multisectoral Action Plan for a Healthy Lifestyle 2015–2020) [172]
Oman	Recommended salt intake: 5 g/day—2019 (MOH) (National Nutrition Strategy 2020–2030, National Plan for Prevention of NCD 2016–2025) [173]
Qatar	Salt intake as per the WHO recommended maximum of 5 g/day for adults—2013 (MOPH) (Part of Nutrition & Physical Activity Action Plan 2011–2016) [180]
Tunisia	Reduce mean salt intake by 30% by 2025 amongst 15 year olds and above—2018 (MOH) (National Multisectoral Strategy for Prevention and Control of NCDs) [86]
UAE	Reduce population salt intake by 30%—2017 (MOHAP) (National Action Plan in Nutrition) [185]

Abbreviations: EMR: Eastern Mediterranean Region; KSA: Kingdom of Saudi Arabia; MOH: Ministry of Health; MOHAP: Ministry of Health and Prevention; MOHP: Ministry of Health and Population; MOPH: Ministry of Public Health; NCDs: non-communicable diseases; SFDA: Saudi Food and Drug Authority; UAE: United Arab Emirates; WHO: World Health Organization.

## Data Availability

Not applicable.

## Supplementary Materials

The following are available online at https://www.mdpi.com/article/10.3390/nu13082676/s1, Table S1: Example of a Database Search, Table S2: Population Salt Intakes in Countries of the EMR.

## Abbreviations

CHD: coronary heart disease; CVD: cardiovascular diseases; DALYs: disability-adjusted life years; EMR: Eastern Mediterranean Region; EMRO: Regional Office for the Eastern Mediterranean; FOP: front of pack; FOPL: front of pack labelling; FFQs: food frequency questionnaires; GBD: Global Burden of Disease; GCC: Gulf Cooperation Council; GINA: Global Database on the Implementation of Nutrition Action; HBP: high blood pressure; KAB: knowledge, attitudes and behavior; KFMBC: Kuwait Flour Mills and Bakeries Company; KSA: Kingdom of Saudi Arabia; LYG: life years gained; MEHE: Ministry of Education and Higher Education; MENA: Middle East and North Africa; MOCI: Ministry of Commerce and Industry; MOH: Ministry of Health; MOHAP: Ministry of Health and Prevention; MOHP: Ministry of Health and Population; MOPH: Ministry of Public Health; MSG: monosodium glutamate; NA: not available; NCD: non-communicable disease; NFP: nutrition focal point; NGO: non-governmental organization; SDGs: Sustainable Development Goals; SFDA: Saudi Food and Drug Authority; UAE: United Arab Emirates; WHO: World Health Organization.

## Appendix A

**Table A1:** List of Search Terms.

Salt	salt* OR *sodium OR Na OR NaCl OR MSG
AND	
Reduction	reduce* OR reduction* OR reducing OR decreas* OR limit OR limits OR limitation* OR limiting OR restrict* OR reformulat* OR low*
OR	
Intake	consumption OR consuming OR consume OR consumes OR intake* OR food* OR nutrition OR diet* OR urinary OR excret*
AND	
Strategy/policy	standard* OR polic* OR initiative* OR tax* OR program* OR regulation* OR strateg* OR guideline* OR practice* OR legislat* OR action* OR plan OR plans OR intervention*
AND	
EMR	Afghan* OR Bahrain* OR Iran* OR Persia* OR Iraq* OR Jordan* OR Kuwait* OR Lebanon* OR Lebanese OR Libya* OR Oman* OR Palestin* OR Gaza* OR “West Bank” OR Qatar* OR Saud* OR KSA OR Syria* OR Tunis* OR “United Arab Emirate*” OR UAE OR Djibouti* OR Egypt* OR Morocc* OR Pakistan* OR Somal* OR Sudan* OR Yemen* OR Levant* OR “East* Mediterranean” OR Gulf OR GCC OR Arab OR Arabia OR Arabs OR EMR OR “Middle East*” OR MENA OR “North* Africa*” OR “East* Africa*” OR “Near East*” OR “Abu Dhabi” OR Dubai OR Ajman OR Fujaira* OR Sharja* OR *Khaima* OR *Qaiwain OR *Quwain

Abbreviations: EMR: Eastern Mediterranean Region; GCC: Gulf Cooperation Council; KSA: Kingdom of Saudi Arabia; MENA: Middle East and North Africa; MSG: monosodium glutamate; UAE: United Arab Emirates.
