# Supplementary material for: Salt Reduction Initiatives in the Eastern Mediterranean Region and Evaluation of Progress towards the 2025 Global Target: A Systematic Review
Source: Nutrients. 2021 Jul 31;13(8):2676. doi: 10.3390/nu13082676 (PMC8399509; doi:10.3390/nu13082676)
Supplement: Supplementary file 1 [file nutrients-13-02676-s001.zip › nutrients-1290385-supplementary.pdf]

nutrients

MDPI

Table S1. Example of a Database Search.

Search Number	Query	Filters	Search Details	Results
7	((((Afghanistan*[tiab] OR Bahrain*[tiab] OR Egypt*[tiab] OR Iran*[tiab] OR Persia*[tiab] OR Iraq*[tiab] OR Jordan*[tiab] OR Kuwait*[tiab] OR Lebanese[tiab] OR Lebanon[tiab] OR Oman*[tiab] OR Palestin*[tiab] OR Gaza*[tiab] OR "West Bank"[tiab] OR Qatar*[tiab] OR Saudi*[tiab] OR KSA[tiab] OR Syria*[tiab] OR "Tunis*"[tiab] OR "United Arab Emirates"[tiab] OR UAE[tiab] OR "Abu Dhabi"[tiab] OR Dubai[tiab] OR Ajman[tiab] OR Fujaira*[tiab] OR Sharja*[tiab] OR Khaima*[tiab] OR Qaiwain[tiab] OR Quwain[tiab] OR Yemen*[tiab] OR Libya*[tiab] OR Djibouti*[tiab] OR Morocc*[tiab] OR Pakistan*[tiab] OR Somalia*[tiab] OR Sudan*[tiab] OR "Middle East"[tiab] OR "Middle Eastern"[tiab] OR Arab[tiab] OR Arabic[tiab] OR Arabs[tiab] OR Arabia[tiab] OR "Near East"[tiab] OR "Near Eastern"[tiab] OR Levant*[tiab] OR MENA[tiab] OR EMR[tiab] OR "East Mediterranean"[tiab] OR "Eastern Mediterranean"[tiab] OR Gulf[tiab] OR GCC[tiab] OR "North Africa"[tiab] OR "North African"[tiab] OR "Northern Africa"[tiab] OR "Northern African"[tiab] OR "East Africa"[tiab] OR "East African"[tiab] OR "Eastern Africa"[tiab] OR "Eastern African"[tiab]) OR ("Africa, Eastern"[Mesh:NoExp] OR "Djibouti"[Mesh] OR "Somalia"[Mesh] OR "South Sudan"[Mesh] OR "Sudan"[Mesh] OR "Africa, Northern"[Mesh:NoExp] OR "Egypt"[Mesh] OR "Libya"[Mesh] OR "Morocco"[Mesh] OR "Tunisia"[Mesh] OR "Middle East"[Mesh:NoExp] OR "Afghanistan"[Mesh] OR "Bahrain"[Mesh] OR "Iran"[Mesh] OR "Iraq"[Mesh] OR "Jordan"[Mesh] OR "Kuwait"[Mesh] OR "Lebanon"[Mesh] OR "Oman"[Mesh] OR "Qatar"[Mesh] OR "Saudi Arabia"[Mesh] OR "Syria"[Mesh] OR "United Arab Emirates"[Mesh] OR "Yemen"[Mesh] OR "Pakistan"[Mesh])) AND ("Tax Exemption"[Mesh] OR "Taxes"[Mesh:NoExp] OR "Government Programs"[Mesh:NoExp] OR "Nutrition Policy"[Mesh] OR "Legislation, Food"[Mesh] OR standard*[tiab] OR polic*[tiab] OR initiative*[tiab] OR tax[tiab] OR taxes[tiab] OR taxation*[tiab] OR taxable[tiab] OR program*[tiab] OR regulation*[tiab] OR strateg*[tiab] OR guideline*[tiab] OR practice*[tiab] OR legislat*[tiab] OR action*[tiab] OR plan[tiab] OR plans[tiab] OR intervention*[tiab])) AND (reduce*[tiab] OR reduction*[tiab] OR reducing[tiab] OR decreas*[tiab] OR limit[tiab] OR limitation*[tiab] OR limiting[tiab] OR restrict*[tiab] OR reformulat*[tiab] OR low[tiab] OR lower*[tiab] OR consumption[tiab] OR	Arabic, English, French, from 1995 - 2021, excluded 56 items related to RTCs (Clinical Trial; Clinical Trial, Phase I; Clinical Trial, Phase II; Clinical Trial, Phase III; Clinical Trial, Phase VI; Clinical Trial Protocol; Controlled Clinical Trial; Pragmatic Clinical Trial; Randomized Controlled Trial; Clinical Trial, Veterinary)	--	883

	consuming[tiab] OR consume[tiab] OR intake*[tiab] OR food*[tiab] OR nutrition[tiab] OR diet*[tiab] OR urinary[tiab] OR excret*[tiab])) AND ("Diet, Sodium-Restricted"[Mesh] OR "Sodium Chloride, Dietary"[Mesh] OR "Sodium, Dietary"[Mesh] OR "Sodium Chloride"[Mesh] OR "Sodium Glutamate"[Mesh] OR salt*[tiab] OR sodium*[tiab] OR Na[tiab] OR NaCl[tiab] OR MSG[tiab])			
6	((((Afghanistan*[tiab] OR Bahrain*[tiab] OR Egypt*[tiab] OR Iran*[tiab] OR Persia*[tiab] OR Iraq*[tiab] OR Jordan*[tiab] OR Kuwait*[tiab] OR Lebanese[tiab] OR Lebanon[tiab] OR Oman*[tiab] OR Palestin*[tiab] OR Gaza*[tiab] OR "West Bank"[tiab] OR Qatar*[tiab] OR Saudi*[tiab] OR KSA[tiab] OR Syria*[tiab] OR "Tunis*"[tiab] OR "United Arab Emirates"[tiab] OR UAE[tiab] OR "Abu Dhabi"[tiab] OR Dubai[tiab] OR Ajman[tiab] OR Fujaira*[tiab] OR Sharja*[tiab] OR Khaima*[tiab] OR Qaiwain[tiab] OR Quwain[tiab] OR Yemen*[tiab] OR Libya*[tiab] OR Djibouti*[tiab] OR Morocc*[tiab] OR Pakistan*[tiab] OR Somalia*[tiab] OR Sudan*[tiab] OR "Middle East"[tiab] OR "Middle Eastern"[tiab] OR Arab[tiab] OR Arabic[tiab] OR Arabs[tiab] OR Arabia[tiab] OR "Near East"[tiab] OR "Near Eastern"[tiab] OR Levant*[tiab] OR MENA[tiab] OR EMR[tiab] OR "East Mediterranean"[tiab] OR "Eastern Mediterranean"[tiab] OR Gulf[tiab] OR GCC[tiab] OR "North Africa"[tiab] OR "North African"[tiab] OR "Northern Africa"[tiab] OR "Northern African"[tiab] OR "East Africa"[tiab] OR "East African"[tiab] OR "Eastern Africa"[tiab] OR "Eastern African"[tiab]) OR ("Africa, Eastern"[Mesh:NoExp] OR "Djibouti"[Mesh] OR "Somalia"[Mesh] OR "South Sudan"[Mesh] OR "Sudan"[Mesh] OR "Africa, Northern"[Mesh:NoExp] OR "Egypt"[Mesh] OR "Libya"[Mesh] OR "Morocco"[Mesh] OR "Tunisia"[Mesh] OR "Middle East"[Mesh:NoExp] OR "Afghanistan"[Mesh] OR "Bahrain"[Mesh] OR "Iran"[Mesh] OR "Iraq"[Mesh] OR "Jordan"[Mesh] OR "Kuwait"[Mesh] OR "Lebanon"[Mesh] OR "Oman"[Mesh] OR "Qatar"[Mesh] OR "Saudi Arabia"[Mesh] OR "Syria"[Mesh] OR "United Arab Emirates"[Mesh] OR "Yemen"[Mesh] OR "Pakistan"[Mesh])) AND ("Tax Exemption"[Mesh] OR "Taxes"[Mesh:NoExp] OR "Government Programs"[Mesh:NoExp] OR "Nutrition Policy"[Mesh] OR "Legislation, Food"[Mesh] OR standard*[tiab] OR polic*[tiab] OR initiative*[tiab] OR tax[tiab] OR taxes[tiab] OR taxation*[tiab] OR taxable[tiab] OR program*[tiab] OR regulation*[tiab] OR strateg*[tiab] OR guideline*[tiab] OR practice*[tiab] OR legislat*[tiab] OR action*[tiab] OR plan[tiab] OR plans[tiab] OR intervention*[tiab])) AND (reduce*[tiab] OR reduction*[tiab] OR reduc-	Arabic, English, French, from 1995 - 2021	("afghanistan*"[Title/Abstract] OR "bahrain*"[Title/Abstract] OR "egypt*"[Title/Abstract] OR "iran*"[Title/Abstract] OR "persia*"[Title/Abstract] OR "iraq*"[Title/Abstract] OR "jordan*"[Title/Abstract] OR "kuwait*"[Title/Abstract] OR "Lebanese"[Title/Abstract] OR "Lebanon"[Title/Abstract] OR "oman*"[Title/Abstract] OR "palestin*"[Title/Abstract] OR "gaza*"[Title/Abstract] OR "West Bank"[Title/Abstract] OR "qatar*"[Title/Abstract] OR "saudi*"[Title/Abstract] OR "KSA"[Title/Abstract] OR "syria*"[Title/Abstract] OR "tunis*"[Title/Abstract]	939

	ing[tiab] OR decreas*[tiab] OR limit[tiab] OR limitation*[tiab] OR limiting[tiab] OR restrict*[tiab] OR reformulat*[tiab] OR low[tiab] OR lower*[tiab] OR consumption[tiab] OR consuming[tiab] OR consume[tiab] OR intake*[tiab] OR food*[tiab] OR nutrition[tiab] OR diet*[tiab] OR urinary[tiab] OR excret*[tiab])) AND ("Diet, Sodium-Restricted"[Mesh] OR "Sodium Chloride, Dietary"[Mesh] OR "Sodium, Dietary"[Mesh] OR "Sodium Chloride"[Mesh] OR "Sodium Glutamate"[Mesh] OR salt*[tiab] OR sodium*[tiab] OR Na[tiab] OR NaCl[tiab] OR MSG[tiab])	OR "United Arab Emirates"[Title/Abstract] OR "UAE"[Title/Abstract] OR "Abu Dhabi"[Title/Abstract] OR "Dubai"[Title/Abstract] OR "Ajman"[Title/Abstract] OR "fujaira*"[Title/Abstract] OR "sharja*"[Title/Abstract] OR "khaima*"[Title/Abstract] OR "Quwain"[Title/Abstract] OR "yemen*"[Title/Abstract] OR "libya*"[Title/Abstract] OR "djibouti*"[Title/Abstract] OR "morocc*"[Title/Abstract] OR "pakistan*"[Title/Abstract] OR "somalia*"[Title/Abstract] OR "sudan*"[Title/Abstract] OR "Middle East"[Title/Abstract] OR "Middle Eastern"[Title/Abstract] OR "Arab"[Title/Abstract] OR "Arabic"[Title/Abstract] OR "Arabs"[Ti-

	tle/Abstract] OR "Arabia"[Title/Abstract] OR "Near East"[Title/Abstract] OR "Near Eastern"[Title/Abstract] OR "levant*"[Title/Abstract] OR "MENA"[Title/Abstract] OR "EMR"[Title/Abstract] OR "East Mediterranean"[Title/Abstract] OR "Eastern Mediterranean"[Title/Abstract] OR "Gulf"[Title/Abstract] OR "GCC"[Title/Abstract] OR "North Africa"[Title/Abstract] OR "North African"[Title/Abstract] OR "Northern Africa"[Title/Abstract] OR "Northern African"[Title/Abstract] OR "East Africa"[Title/Abstract] OR "East African"[Title/Abstract] OR "Eastern Africa"[Title/Abstract] OR "Eastern African"[Title/Abstract] OR ("africa, east-

		ern"[MeSH Terms:noexp] OR "Djibouti"[MeSH Terms] OR "Somalia"[MeSH Terms] OR "South Sudan"[MeSH Terms] OR "Sudan"[MeSH Terms] OR "africa, northern"[MeSH Terms:noexp] OR "Egypt"[MeSH Terms] OR "Libya"[MeSH Terms] OR "Morocco"[MeSH Terms] OR "Tunisia"[MeSH Terms] OR "Middle East"[MeSH Terms:noexp] OR "Afghanistan"[MeSH Terms] OR "Bahrain"[MeSH Terms] OR "Iran"[MeSH Terms] OR "Iraq"[MeSH Terms] OR "Jordan"[MeSH Terms] OR "Kuwait"[MeSH Terms] OR "Lebanon"[MeSH Terms] OR "Oman"[MeSH Terms] OR "Qatar"[MeSH Terms] OR "Saudi Arabia"[MeSH Terms] OR "Syria"[MeSH Terms]

	OR "United Arab Emirates"[MeSH Terms] OR "Yemen"[MeSH Terms] OR "Pakistan"[MeSH Terms])) AND ("Tax Exemption"[MeSH Terms] OR "Taxes"[MeSH Terms:noexp] OR "Government Programs"[MeSH Terms:noexp] OR "Nutrition Policy"[MeSH Terms] OR "legislation, food"[MeSH Terms] OR "standard*"[Title/Abstract] OR "polic*"[Title/Abstract] OR "initiative*"[Title/Abstract] OR "tax"[Title/Abstract] OR "Taxes"[Title/Abstract] OR "taxation*"[Title/Abstract] OR "taxable"[Title/Abstract] OR "program*"[Title/Abstract] OR "regulation*"[Title/Abstract] OR "strateg*"[Title/Abstract] OR "guideline*"[Title/Abstract]

OR "practice*"[Title/Abstract] OR "legislat*"[Title/Abstract] OR "action*"[Title/Abstract] OR "plan"[Title/Abstract] OR "plans"[Title/Abstract] OR "intervention*"[Title/Abstract]) AND ("reduce*"[Title/Abstract] OR "reduction*"[Title/Abstract] OR "reducing"[Title/Abstract] OR "decreas*"[Title/Abstract] OR "limit"[Title/Abstract] OR "limitation*"[Title/Abstract] OR "limiting"[Title/Abstract] OR "restrict*"[Title/Abstract] OR "reformulat*"[Title/Abstract] OR "low"[Title/Abstract] OR "lower*"[Title/Abstract] OR "consumption"[Title/Abstract] OR "consuming"[Title/Abstract] OR "consume"[Title/Abstract] OR "intake*"[Title/Ab-

			stract] OR "food*"[Title/Abstract] OR "nutrition"[Title/Abstract] OR "diet*"[Title/Abstract] OR "urinary"[Title/Abstract] OR "excret*"[Title/Abstract]) AND ("diet, sodium restricted"[MeSH Terms] OR "sodium chloride, dietary"[MeSH Terms] OR "sodium, dietary"[MeSH Terms] OR "Sodium Chloride"[MeSH Terms] OR "Sodium Glutamate"[MeSH Terms] OR "salt*"[Title/Abstract] OR "sodium*"[Title/Abstract] OR "Na"[Title/Abstract] OR "NaCl"[Title/Abstract] OR "MSG"[Title/Abstract])	
5	((((Afghanistan*[tiab] OR Bahrain*[tiab] OR Egypt*[tiab] OR Iran*[tiab] OR Persia*[tiab] OR Iraq*[tiab] OR Jordan*[tiab] OR Kuwait*[tiab] OR Lebanese[tiab] OR Lebanon[tiab] OR Oman*[tiab] OR Palestin*[tiab] OR Gaza*[tiab] OR "West Bank"[tiab] OR Qatar*[tiab] OR Saudi*[tiab] OR KSA[tiab] OR Syria*[tiab] OR "Tunis*"[tiab] OR "United Arab Emirates"[tiab] OR UAE[tiab] OR "Abu Dhabi"[tiab] OR Dubai[tiab] OR Ajman[tiab] OR Fujaira*[tiab] OR Sharja*[tiab] OR Khaima*[tiab] OR Qaiwain[tiab] OR Quwain[tiab] OR Yemen*[tiab] OR Libya*[tiab] OR Djibouti*[tiab] OR Morocc*[tiab] OR Pakistan*[tiab] OR Somalia*[tiab] OR Sudan*[tiab] OR "Middle East"[tiab] OR "Middle Eastern"[tiab] OR	--	("afghanistan*"[Title/Abstract] OR "bahrain*"[Title/Abstract] OR "egypt*"[Title/Abstract] OR "iran*"[Title/Abstract] OR "persia*"[Title/Abstract] OR "iraq*"[Title/Abstract]	1,042

	Arab[tiab] OR Arabic[tiab] OR Arabs[tiab] OR Arabia[tiab] OR "Near East"[tiab] OR "Near Eastern"[tiab] OR Levant*[tiab] OR MENA[tiab] OR EMR[tiab] OR "East Mediterranean"[tiab] OR "Eastern Mediterranean"[tiab] OR Gulf[tiab] OR GCC[tiab] OR "North Africa"[tiab] OR "North African"[tiab] OR "Northern Africa"[tiab] OR "Northern African"[tiab] OR "East Africa"[tiab] OR "East African"[tiab] OR "Eastern Africa"[tiab] OR "Eastern African"[tiab]) OR ("Africa, Eastern"[Mesh:NoExp] OR "Djibouti"[Mesh] OR "Somalia"[Mesh] OR "South Sudan"[Mesh] OR "Sudan"[Mesh] OR "Africa, Northern"[Mesh:NoExp] OR "Egypt"[Mesh] OR "Libya"[Mesh] OR "Morocco"[Mesh] OR "Tunisia"[Mesh] OR "Middle East"[Mesh:NoExp] OR "Afghanistan"[Mesh] OR "Bahrain"[Mesh] OR "Iran"[Mesh] OR "Iraq"[Mesh] OR "Jordan"[Mesh] OR "Kuwait"[Mesh] OR "Lebanon"[Mesh] OR "Oman"[Mesh] OR "Qatar"[Mesh] OR "Saudi Arabia"[Mesh] OR "Syria"[Mesh] OR "United Arab Emirates"[Mesh] OR "Yemen"[Mesh] OR "Pakistan"[Mesh])) AND ("Tax Exemption"[Mesh] OR "Taxes"[Mesh:NoExp] OR "Government Programs"[Mesh:NoExp] OR "Nutrition Policy"[Mesh] OR "Legislation, Food"[Mesh] OR standard*[tiab] OR polic*[tiab] OR initiative*[tiab] OR tax[tiab] OR taxes[tiab] OR taxation*[tiab] OR taxable[tiab] OR program*[tiab] OR regulation*[tiab] OR strateg*[tiab] OR guideline*[tiab] OR practice*[tiab] OR legislat*[tiab] OR action*[tiab] OR plan[tiab] OR plans[tiab] OR intervention*[tiab])) AND (reduce*[tiab] OR reduction*[tiab] OR reducing[tiab] OR decreas*[tiab] OR limit[tiab] OR limitation*[tiab] OR limiting[tiab] OR restrict*[tiab] OR reformulat*[tiab] OR low[tiab] OR lower*[tiab] OR consumption[tiab] OR consuming[tiab] OR consume[tiab] OR intake*[tiab] OR food*[tiab] OR nutrition[tiab] OR diet*[tiab] OR urinary[tiab] OR excret*[tiab])) AND ("Diet, Sodium-Restricted"[Mesh] OR "Sodium Chloride, Dietary"[Mesh] OR "Sodium, Dietary"[Mesh] OR "Sodium Chloride"[Mesh] OR "Sodium Glutamate"[Mesh] OR salt*[tiab] OR sodium*[tiab] OR Na[tiab] OR NaCl[tiab] OR MSG[tiab])	OR "jordan*"[Title/Abstract] OR "kuwait*"[Title/Abstract] OR "Lebanese"[Title/Abstract] OR "Lebanon"[Title/Abstract] OR "oman*"[Title/Abstract] OR "palestin*"[Title/Abstract] OR "gaza*"[Title/Abstract] OR "West Bank"[Title/Abstract] OR "qatar*"[Title/Abstract] OR "saudi*"[Title/Abstract] OR "KSA"[Title/Abstract] OR "syria*"[Title/Abstract] OR "tunis*"[Title/Abstract] OR "United Arab Emirates"[Title/Abstract] OR "UAE"[Title/Abstract] OR "Abu Dhabi"[Title/Abstract] OR "Dubai"[Title/Abstract] OR "Ajman"[Title/Abstract] OR "fujaira*"[Title/Abstract] OR "sharja*"[Title/Abstract] OR "khaima*"[Title/Abstract] OR

	"Quwain"[Title/Abstract] OR "yemen*"[Title/Abstract] OR "libya*"[Title/Abstract] OR "djibouti*"[Title/Abstract] OR "morocc*"[Title/Abstract] OR "pakistan*"[Title/Abstract] OR "somalia*"[Title/Abstract] OR "sudan*"[Title/Abstract] OR "Middle East"[Title/Abstract] OR "Middle Eastern"[Title/Abstract] OR "Arab"[Title/Abstract] OR "Arabic"[Title/Abstract] OR "Arabs"[Title/Abstract] OR "Arabia"[Title/Abstract] OR "Near East"[Title/Abstract] OR "Near Eastern"[Title/Abstract] OR "levant*"[Title/Abstract] OR "MENA"[Title/Abstract] OR "EMR"[Title/Abstract] OR "East Mediterranean"[Title/Abstract] OR "Eastern Mediterranean"[Title/Abstract]

	OR "Gulf"[Title/Abstract] OR "GCC"[Title/Abstract] OR "North Africa"[Title/Abstract] OR "North African"[Title/Abstract] OR "Northern Africa"[Title/Abstract] OR "Northern African"[Title/Abstract] OR "East Africa"[Title/Abstract] OR "East African"[Title/Abstract] OR "Eastern Africa"[Title/Abstract] OR "Eastern African"[Title/Abstract] OR ("africa, eastern"[MeSH Terms:noexp] OR "Djibouti"[MeSH Terms] OR "Somalia"[MeSH Terms] OR "South Sudan"[MeSH Terms] OR "Sudan"[MeSH Terms] OR "africa, northern"[MeSH Terms:noexp] OR "Egypt"[MeSH Terms] OR "Libya"[MeSH Terms] OR "Morocco"[MeSH	

	Terms] OR "Tunisia"[MeSH Terms] OR "Middle East"[MeSH Terms:noexp] OR "Afghanistan"[MeSH Terms] OR "Bahrain"[MeSH Terms] OR "Iran"[MeSH Terms] OR "Iraq"[MeSH Terms] OR "Jordan"[MeSH Terms] OR "Kuwait"[MeSH Terms] OR "Lebanon"[MeSH Terms] OR "Oman"[MeSH Terms] OR "Qatar"[MeSH Terms] OR "Saudi Arabia"[MeSH Terms] OR "Syria"[MeSH Terms] OR "United Arab Emirates"[MeSH Terms] OR "Yemen"[MeSH Terms] OR "Pakistan"[MeSH Terms])) AND ("Tax Exemption"[MeSH Terms] OR "Taxes"[MeSH Terms:noexp] OR "Government Programs"[MeSH Terms:noexp] OR "Nutrition Policy"[MeSH

	Terms] OR "legislation, food"[MeSH Terms] OR "standard*"[Title/Abstract] OR "polic*"[Title/Abstract] OR "initiative*"[Title/Abstract] OR "tax"[Title/Abstract] OR "Taxes"[Title/Abstract] OR "taxation*"[Title/Abstract] OR "taxable"[Title/Abstract] OR "program*"[Title/Abstract] OR "regulation*"[Title/Abstract] OR "strateg*"[Title/Abstract] OR "guideline*"[Title/Abstract] OR "practice*"[Title/Abstract] OR "legislat*"[Title/Abstract] OR "action*"[Title/Abstract] OR "plan"[Title/Abstract] OR "plans"[Title/Abstract] OR "intervention*"[Title/Abstract]) AND ("reduce*"[Title/Abstract] OR "reduction*"[Title/Abstract]

	OR "reducing"[Title/Abstract] OR "decreas*"[Title/Abstract] OR "limit"[Title/Abstract] OR "limitation*"[Title/Abstract] OR "limiting"[Title/Abstract] OR "restrict*"[Title/Abstract] OR "reformulat*"[Title/Abstract] OR "low"[Title/Abstract] OR "lower*"[Title/Abstract] OR "consumption"[Title/Abstract] OR "consuming"[Title/Abstract] OR "consume"[Title/Abstract] OR "intake*"[Title/Abstract] OR "food*"[Title/Abstract] OR "nutrition"[Title/Abstract] OR "diet*"[Title/Abstract] OR "urinary"[Title/Abstract] OR "excret*"[Title/Abstract]) AND ("diet, sodium restricted"[MeSH Terms] OR "sodium chloride, dietary"[MeSH Terms] OR "sodium, dietary"[MeSH Terms] OR

			"Sodium Chloride"[MeSH Terms] OR "Sodium Glutamate"[MeSH Terms] OR "salt*"[Title/Abstract] OR "sodium*"[Title/Abstract] OR "Na"[Title/Abstract] OR "NaCl"[Title/Abstract] OR "MSG"[Title/Abstract])	
4	(Afghanistan*[tiab] OR Bahrain*[tiab] OR Egypt*[tiab] OR Iran*[tiab] OR Persia*[tiab] OR Iraq*[tiab] OR Jordan*[tiab] OR Kuwait*[tiab] OR Lebanese[tiab] OR Lebanon[tiab] OR Oman*[tiab] OR Palestin*[tiab] OR Gaza*[tiab] OR "West Bank"[tiab] OR Qatar*[tiab] OR Saudi*[tiab] OR KSA[tiab] OR Syria*[tiab] OR "Tunis*"[tiab] OR "United Arab Emirates"[tiab] OR UAE[tiab] OR "Abu Dhabi"[tiab] OR Dubai[tiab] OR Ajman[tiab] OR Fujaira*[tiab] OR Sharja*[tiab] OR Khaima*[tiab] OR Qaiwain[tiab] OR Quwain[tiab] OR Yemen*[tiab] OR Libya*[tiab] OR Djibouti*[tiab] OR Morocc*[tiab] OR Pakistan*[tiab] OR Somalia*[tiab] OR Sudan*[tiab] OR "Middle East"[tiab] OR "Middle Eastern"[tiab] OR Arab[tiab] OR Arabic[tiab] OR Arabs[tiab] OR Arabia[tiab] OR "Near East"[tiab] OR "Near Eastern"[tiab] OR Levant*[tiab] OR MENA[tiab] OR EMR[tiab] OR "East Mediterranean"[tiab] OR "Eastern Mediterranean"[tiab] OR Gulf[tiab] OR GCC[tiab] OR "North Africa"[tiab] OR "North African"[tiab] OR "Northern Africa"[tiab] OR "Northern African"[tiab] OR "East Africa"[tiab] OR "East African"[tiab] OR "Eastern Africa"[tiab] OR "Eastern African"[tiab]) OR ("Africa, Eastern"[Mesh:NoExp] OR "Djibouti"[Mesh] OR "Somalia"[Mesh] OR "South Sudan"[Mesh] OR "Sudan"[Mesh] OR "Africa, Northern"[Mesh:NoExp] OR "Egypt"[Mesh] OR "Libya"[Mesh] OR "Morocco"[Mesh] OR "Tunisia"[Mesh] OR "Middle East"[Mesh:NoExp] OR "Afghanistan"[Mesh] OR "Bahrain"[Mesh] OR "Iran"[Mesh] OR "Iraq"[Mesh] OR "Jordan"[Mesh] OR "Kuwait"[Mesh] OR "Lebanon"[Mesh] OR "Oman"[Mesh] OR "Qatar"[Mesh] OR "Saudi Arabia"[Mesh] OR "Syria"[Mesh] OR "United Arab Emirates"[Mesh] OR "Yemen"[Mesh] OR "Pakistan"[Mesh])	--	"afghanistan*"[Title/Abstract] OR "bahrain*"[Title/Abstract] OR "egypt*"[Title/Abstract] OR "iran*"[Title/Abstract] OR "persia*"[Title/Abstract] OR "iraq*"[Title/Abstract] OR "jordan*"[Title/Abstract] OR "kuwait*"[Title/Abstract] OR "Lebanese"[Title/Abstract] OR "Lebanon"[Title/Abstract] OR "oman*"[Title/Abstract] OR "palestin*"[Title/Abstract] OR "gaza*"[Title/Abstract] OR "West Bank"[Title/Abstract] OR "qatar*"[Title/Abstract] OR	288,017

	"saudi*"[Title/Abstract] OR "KSA"[Title/Abstract] OR "syria*"[Title/Abstract] OR "tunis*"[Title/Abstract] OR "United Arab Emirates"[Title/Abstract] OR "UAE"[Title/Abstract] OR "Abu Dhabi"[Title/Abstract] OR "Dubai"[Title/Abstract] OR "Ajman"[Title/Abstract] OR "fujaira*"[Title/Abstract] OR "sharja*"[Title/Abstract] OR "khaima*"[Title/Abstract] OR "Quwain"[Title/Abstract] OR "yemen*"[Title/Abstract] OR "libya*"[Title/Abstract] OR "djibouti*"[Title/Abstract] OR "morocc*"[Title/Abstract] OR "pakistan*"[Title/Abstract] OR "somalia*"[Title/Abstract] OR "sudan*"[Title/Abstract] OR "Middle East"[Title/Abstract]

	OR "Middle Eastern"[Title/Abstract] OR "Arab"[Title/Abstract] OR "Arabic"[Title/Abstract] OR "Arabs"[Title/Abstract] OR "Arabia"[Title/Abstract] OR "Near East"[Title/Abstract] OR "Near Eastern"[Title/Abstract] OR "levant*"[Title/Abstract] OR "MENA"[Title/Abstract] OR "EMR"[Title/Abstract] OR "East Mediterranean"[Title/Abstract] OR "Eastern Mediterranean"[Title/Abstract] OR "Gulf"[Title/Abstract] OR "GCC"[Title/Abstract] OR "North Africa"[Title/Abstract] OR "North African"[Title/Abstract] OR "Northern Africa"[Title/Abstract] OR "Northern African"[Title/Abstract] OR "East Africa"[Title/Abstract] OR "East African"[Ti-

	tle/Abstract] OR "Eastern Africa"[Title/Abstract] OR "Eastern African"[Title/Abstract] OR "africa, eastern"[MeSH Terms:noexp] OR "Djibouti"[MeSH Terms] OR "Somalia"[MeSH Terms] OR "South Sudan"[MeSH Terms] OR "Sudan"[MeSH Terms] OR "africa, northern"[MeSH Terms:noexp] OR "Egypt"[MeSH Terms] OR "Libya"[MeSH Terms] OR "Morocco"[MeSH Terms] OR "Tunisia"[MeSH Terms] OR "Middle East"[MeSH Terms:noexp] OR "Afghanistan"[MeSH Terms] OR "Bahrain"[MeSH Terms] OR "Iran"[MeSH Terms] OR "Iraq"[MeSH Terms] OR "Jordan"[MeSH Terms] OR "Kuwait"[MeSH Terms] OR "Lebanon"[MeSH Terms] OR	

			"Oman"[MeSH Terms] OR "Qatar"[MeSH Terms] OR "Saudi Arabia"[MeSH Terms] OR "Syria"[MeSH Terms] OR "United Arab Emirates"[MeSH Terms] OR "Yemen"[MeSH Terms] OR "Pakistan"[MeSH Terms]	
3	"Tax Exemption"[Mesh] OR "Taxes"[Mesh:NoExp] OR "Government Programs"[Mesh:NoExp] OR "Nutrition Policy"[Mesh] OR "Legislation, Food"[Mesh] OR standard*[tiab] OR polic*[tiab] OR initiative*[tiab] OR tax[tiab] OR taxes[tiab] OR taxation*[tiab] OR taxable[tiab] OR program*[tiab] OR regulation*[tiab] OR strateg*[tiab] OR guideline*[tiab] OR practice*[tiab] OR legislat*[tiab] OR action*[tiab] OR plan[tiab] OR plans[tiab] OR intervention*[tiab]	--	"Tax Exemption"[MeSH Terms] OR "Taxes"[MeSH Terms:noexp] OR "Government Programs"[MeSH Terms:noexp] OR "Nutrition Policy"[MeSH Terms] OR "legislation, food"[MeSH Terms] OR "standard*"[Title/Abstract] OR "polic*"[Title/Abstract] OR "initiative*"[Title/Abstract] OR "tax"[Title/Abstract] OR "Taxes"[Title/Abstract] OR "taxation*"[Title/Abstract] OR "taxable"[Title/Abstract] OR "program*"[Title/Abstract]	6,379,826

			OR "regulation*"[Title/Abstract] OR "strateg*"[Title/Abstract] OR "guideline*"[Title/Abstract] OR "practice*"[Title/Abstract] OR "legislat*"[Title/Abstract] OR "action*"[Title/Abstract] OR "plan"[Title/Abstract] OR "plans"[Title/Abstract] OR "intervention*"[Title/Abstract]	
2	reduce*[tiab] OR reduction*[tiab] OR reducing[tiab] OR decreas*[tiab] OR limit[tiab] OR limitation*[tiab] OR limiting[tiab] OR restrict*[tiab] OR reformulat*[tiab] OR low[tiab] OR lower*[tiab] OR consumption[tiab] OR consuming[tiab] OR consume[tiab] OR intake*[tiab] OR food*[tiab] OR nutrition[tiab] OR diet*[tiab] OR urinary[tiab] OR excret*[tiab]	--	"reduce*"[Title/Abstract] OR "reduction*"[Title/Abstract] OR "reducing"[Title/Abstract] OR "decreas*"[Title/Abstract] OR "limit"[Title/Abstract] OR "limitation*"[Title/Abstract] OR "limiting"[Title/Abstract] OR "restrict*"[Title/Abstract] OR "reformulat*"[Title/Abstract] OR "low"[Title/Abstract] OR "lower*"[Title/Abstract] OR "consumption"[Title/Abstract]	9,704,384

			OR "consuming"[Title/Abstract] OR "consume"[Title/Abstract] OR "intake*"[Title/Abstract] OR "food*"[Title/Abstract] OR "nutrition"[Title/Abstract] OR "diet*"[Title/Abstract] OR "urinary"[Title/Abstract] OR "excret*"[Title/Abstract]	
1	"Diet, Sodium-Restricted"[Mesh] OR "Sodium Chloride, Dietary"[Mesh] OR "Sodium, Dietary"[Mesh] OR "Sodium Chloride"[Mesh] OR "Sodium Glutamate"[Mesh] OR salt*[tiab] OR sodium*[tiab] OR Na[tiab] OR NaCl[tiab] OR MSG[tiab]	--	"diet, sodium restricted"[MeSH Terms] OR "sodium chloride, dietary"[MeSH Terms] OR "sodium, dietary"[MeSH Terms] OR "Sodium Chloride"[MeSH Terms] OR "Sodium Glutamate"[MeSH Terms] OR "salt*"[Title/Abstract] OR "sodium*"[Title/Abstract] OR "Na"[Title/Abstract] OR "NaCl"[Title/Abstract] OR "MSG"[Title/Abstract]	728,051

Table S2. Population Salt Intakes in Countries of the EMR.

Country	Reference	Year	National or Regional	Method used	Study Population	Estimated salt/sodium intake
**Bahrain**	MOH-Bahrain National Survey [1]	1998-1999	National	FFQ + 24-hr recalls	**Adults aged 19 years and above**	***Mean Na intake (g/day):***<br>Males: 5.3<br>Females: 3.7
**Egypt**	Central Agency for Public Mobilization and Statistics-MOHAP-WHO [2]<br><br>**STEPs survey**	2017-2018	National	Spot urine	**Adults aged 15-69 years**	***Mean salt intake (g/day):***<br>Total: 8.9<br>Males: 9.5<br>Females: 8.1
	AbdEl Aal et al 2018 [3]	--	National	24-hr Na urinary excretion + FFQ + 24-hr recall	--	***Mean Na intake (mg/day):***<br>3243.3 ± 1282.7
	Brouzes et al 2020 [4]	2016-2017	Regional; Greater Cairo, Alexandria, Delta, and Upper Egypt	4 days food diary	**Urban women aged 19-30 years;**<br>N=130	***Mean Na intake (mg/day):***<br>2787 ± 1065
	Zaghloul et al 2018 [5]	2014-2016	Regional; General Organization of Teaching Hospital and Institutes	24-hr recall	**Adult employees aged 25-64 years**;<br>N=96	***Mean Na intake (mg/day):***<br>Males: 3841.4<br>Females: 2735
	Tayel et al 2013 [6]<br><br>**Cross-sectional; random selection**	2012	Regional; Some faculties in Alexandria University	Pre-designed structured interview questionnaire	**University female students aged 18-25 years;**<br>N=300	***Mean Na intake (g/day):***<br>3.67 ± 1.09
	Egypt MAP-NCD 2017 [7]	2010	National	--	**Population aged 18 years and above**	***Mean salt intake (g/day):***<br>12.8
**Iran**	Gholami et al 2020 [8]	2016	National	Spot urine	**Adults aged 25 years and above;**	***Mean salt intake (g/day):***<br>9.3 ± 2.3

**Based on the STEPs cross-sectional survey**				N=8073 (normotensive)	
Razaei et al 2018 [9] and MOHME-Tehran University of Medical Sciences-National Institute of Health Research [10]<br><br>**STEPs survey**	2016	National	Spot urine for all individuals + 24-hr Na urinary excretion for a subsample	**Adults aged 25 years and above;** N=18624	***Mean salt intake (g/day):***<br>Total: 9.52<br>Males: 11<br>Females: 8.25<br>Rural: 9.5<br>Urban: 9
Khosravi et al 2012 (2 articles) [11,12]<br><br>**Cross-sectional, multi-stage cluster sampling**	1999-2000<br>2001-2002<br>2007	Regional; Isfahan	24-hr Na urinary excretion + urinary creatinine	**Adults aged 20-60 years** in 1999-2000; N=1059<br><br>**Adults aged 19 years and above** in 2001-2002; N=374<br><br>**Adults aged 19 years and above** in 2007; N=806	***Mean salt intake (g/day):***<br>1999-2000: 9.1<br>2001-2002: 13.9<br>2007: 11.8
Arsang-Jang et al 2019 [13] and Mohammadifard et al 2019 [14]<br><br>**Isfahan Salt Study; trend analysis on repeated cross-sectional data**	2001-2013	Regional; Isfahan	24-hr urine	**Adults aged 18 years and above;** N=1503	***Mean salt intakes (g/day):***<br>**Year 2001:**<br>Total: 9.7<br>Males: 9.7<br>Females: 9.8<br><br>**Year 2007:**<br>Total: 9.6<br>Males: 10.3

					Females: 9.2<br><br>**Year 2013:**<br>Total: 10.2<br>Males: 11.3<br>Females: 9.3
Motlagh et al 2011 [15]<br><br>**Descriptive, analytical, cross-sectional**	--	Regional; Yazd	24-hr urine	**Adults aged 18-45 years, referred to medical health centers;**<br>N=247	***Mean salt intake (g/day):***<br>10.09 ± 2.97
Mirzaei et al 2014 [16]<br><br>**Cross-sectional; part of the Yazd Healthy Heart project**	2004-2005	Regional; Yazd	24-hr Na urinary excretion	**Adult urban dwellers aged 20-70 years;**<br>N=219 volunteers	***Mean salt intake (g/day):***<br>Males: 10.0 ± 4.8<br>Females: 7.5 ± 3.3
Emamian et al 2021 [17]<br><br>**Based on Shahroud schoolchildren eye cohort study; random cluster sampling**	2015	Regional; Shahroud	Spot urine + creatinine excretion	**Schoolchildren aged 9-15 years**;<br>N=1455	***Mean salt intake (g/day):***<br>Total: 9.7 ± 2.6<br>Males: 9.8 ± 2.6<br>Females: 9.3 ± 2.6
Kelishadi et al 2013 [18]<br><br>**Cross-sectional; multistage cluster sampling**	2011-2012	Regional; Isfahan	Fasting urine	**Children aged 3-10 years;**<br>N=220	***Mean Na intake (mg/day):***<br>2017.76 ± 117.94

Mohammadifard et al 2017 [19]<br><br>**Cross-sectional; multi-stage cluster sampling**	2013-2014	Regional; Isfahan	24-hr urine with urinary creatinine + semi-quantitative FFQ	**Adults aged 18 years and above**;<br>N=796	***Mean Na intake (mg/day):***<br>4309.6 ± 1344.4<br><br>***Mean Na urinary excretion (mg/day):***<br>4069.6 ± 1655.3
Rafiei et al 2008 [20]<br><br>**Experimental, population-based**	2001	Regional; Isfahan	24-hr urine with urinary creatinine + food records	**Adults aged 20-60 years in urban areas**;<br>N=912	***Mean salt intake (g/day):***<br>Total: 9.9 + 2.9<br>Men: 11.1 + 3.0<br>Women: 9.6 + 2.9
Karimi et al 2020 [21]<br><br>**Cross-sectional**	--	National	FFQ	**Children and adolescents aged 6-18 years;**<br>N=5187	***Mean sodium intakes (mg/day):***<br>Boys: 5707.4<br>Girls: 6491.9<br><br><u>By age group:</u><br>6-12 years: 5649.41<br>13-18 years: 6760.1
Mirmiran et al 2019 [22]<br><br>**Cohort; Tehran Lipid and Glucose Study**	2006-2008	Regional; Tehran	Semi-quantitative FFQ	**Children and adolescents aged 6-18 years**;<br>N=424	***Mean salt intake (g/day):***<br>Boys: 11.9 ± 7.69<br>Girls: 12.2 ± 9.9
Shahriarpour et al 2020 [23]<br><br>**Cross-sectional**	--	Regional; Tehran	Semi-quantitative FFQ	**Postmenopausal women aged 50-85 years** in an outpatient health center;<br>N=151	***Mean Na intake (g/day):***<br>2.0 ± 1.2
Azizi et al 2001 [24]	1998	Regional; Rasht and Sari	FFQ	**Population aged 2 years and above**;	***Mean salt intake (g/day):***<br>Rasht: 7.2<br>Sari: 7.7

	**Cross-sectional, cluster sampling**				N=340 in Rasht and 343 in Sari	
	Rahmani et al 2000 [25]<br><br>**Cross-sectional; cluster sampling**	2000	Regional; Ilam	FFQ	**Population aged 2-79 years from 75-61 households;**<br>N=644	***Mean salt intake (g/day):***<br>10.3
	Kooshki and Golafrooz 2009 [26]<br><br>**Descriptive, analytical; cluster sampling**	--	Regional; Sabzevar	24-hr recalls; 3 successive days	**Elderly;**<br>N=100	***Mean Na intake (mg/day):***<br>3993.55 ± 1197
**Iraq**	MOH-WHO [27]<br><br>**STEPs survey**	2015	National	24-hr urine	**Adults aged 18 years and above**	***Mean salt urinary excretion (mmol/24 hrs):***<br>Total: 150.6<br>Males: 155.6<br>Females: 142.6
**Jordan**	Alawwa et al 2018 [28]<br><br>**Cross-sectional; descriptive**	2014	Regional; University of Jordan and the Jordan University Hospital	24-hr urine+ urinary creatinine	Convenient sample of **adults aged 18-64 years**;<br>N=103	***Mean Na intake (g/day):***<br>Total: 4.1<br>Males: 4.3<br>Females: 4
	MOH [29]<br><br>**STEPs Survey**	2019	Regional; Amman	Spot urine	**Jordanian and Syrian adults aged 18-69 years**	***Mean salt intake (g/day):***<br>Total: 11<br>Males: 12.5<br>Females: 9.6
	Al-Wa'l and Takruri 2016 [30]<br><br>**Convenient sample**	--	Regional; University of Jordan	3-day food records	**Students and employees aged 20-40 years**;<br>N=200	***Mean Na intake (mg/day):***<br>Total: 5176<br>Males: 5464<br>Females: 4926
	Alkurd 2011 [31]	2006-2007	National	Based on the Jordanian	N=12768 households	***Daily per capita intake of Na (mg):***<br>Amman: 7062<br>Balqa: 10877

	**Two-stage cluster stratified sampling**			Household Expenditures and Income Survey (JHEIS); questionnaire on expenditure on different foods		Zarqa: 6767<br>Madaba: 8896<br>Irbid: 9155<br>Mafraq: 6556<br>Jarash: 8537<br>Ajloun: 9600<br>Karak: 4747<br>Tafilah: 6281<br>Ma'an: 7232<br>Aqaba: 5762<br>**Entire country: 7623**
	Takruri and Alkurd 2014 [32]<br><br>**Two-stage cluster stratified sampling**	2010	National	Based on the Jordanian Household Expenditures and Income Survey (JHEIS); questionnaire on expenditure on different foods	N=13866	***Mean Na intake (mg/day):***<br>Amman: 5926<br>Balqa: 4589<br>Zarqa: 7004<br>Madaba: 9865<br>Irbid: 5397<br>Mafraq: 6550<br>Jarash: 15614<br>Ajloun: 5480<br>Karak: 8022<br>Tafilah: 7637<br>Ma'an: 6632<br>Aqaba: 7356<br>**Entire country: 6478**
**KSA**	Alkhunaizi et al 2013 [33]	2009-2012	Regional; Eastern KSA	24-hr Na urinary excretion + urinary creatinine	**14 years and above;** N=130<br><br>Samples collected for 4 consecutive years	***Mean Na intake (g/day):***<br>Total: 3.2<br>Males: 3.5<br>Females: 2.7

	Al-Khathaami et al 2019 [34]<br><br>**Cross-sectional, descriptive**	2016	Regional; King Saud bin Abdulaziz University for Health Sciences, Riyadh	Validated electronic FFQ; self-administered	**Undergraduates aged 18 years and above;**<br>N=3522	***Mean salt intake (g/day):***<br>Total: 6.76 ± 3.89<br>Males: 6.92 ± 3.67<br>Females: 6.63 ± 4.08
**Kuwait**	Zaghloul et al 2013 [35]<br><br>**Cross-sectional, multistage stratified, cluster sampling**<br><br>**National Nutrition Survey**	2008-2009	National	24-hr recall	**Kuwaitis aged 3-86 years;**<br>N=1704	***Mean Na intakes (mg/day) by age groups:***<br><u>1-3 years:</u><br>Males: 1607.6 ± 153.3<br>Females: 2038.5 ± 203.9<br><br><u>4-8 years:</u><br>Males: 2610.7 ± 118.7<br>Females: 2339.8 ± 98.5<br><br><u>9-13 years:</u><br>Males: 3508.2 ± 172.4<br>Females: 2975.7 ± 175.7<br><br><u>14-18 years:</u><br>Males: 3679.3 ± 184.8<br>Females: 2652.6 ± 140<br><br><u>19-50 years:</u><br>Males: 3950.3 ± 130.4<br>Females: 2857.7 ± 105.3<br><br><u>Above 50 years:</u><br>Males: 3083.4 ± 153.4<br>Females: 2353.8 ± 103.4
	Alomirah et al 2008 [36]	2008	National	Dietary survey	**Adults**	***Mean Na intake (mg/day):*** 4000 (equivalent to 10 g of salt)

	Public Authority for Food and Nutrition 2020 [37] and WHO 2014 [38]	2014	National	Questionnaire	**Adults aged 18–69 years**	9-11 g of salt/day [37]<br><br>12-15 g of salt/day [38]
	Merhi 2017-Thesis [39]<br><br>**Cross-sectional; cluster sampling**	2013-2014	National	Non-fasting urine + creatinine excretion	**Children aged 6-10 years from schools;** N=1403	***Mean Na intake:***<br>2893.8 ± 726.1 mg/day (equivalent to 5.6 ± 3.63 g/day of salt)
	Nasreddine et al 2014 (unpublished) [40]	--	Regional; Beirut	24-hr Na urinary excretion	**Adults**	***Mean Na intake (g/day):***<br>Total: 3.6<br>Males: 4.8<br>Females: 3.1
**Lebanon**	Choucair 2016 – Thesis [41]<br><br>**Convenience sample**	--	Regional; American University of Beirut and its Medical Center in Beirut	24-hr Na urinary excretion and urinary creatinine + spot urine + FFQ	**Adults aged 19-55 years;** N=60	***Mean Na intake (mg/day):***<br>Based on FFQ:<br>Total: 4968.78 ± 2374.08<br>Males: 5990.18 ± 2003.32<br>Females: 3876.95 ± 2277.44<br><br>Based on spot urine-Kawasaki:<br>Total: 4437.2 ± 1511.45<br>Males: 4927.26 ± 1682.32<br>Females: 3913.34 ± 1109.62<br><br>Based on spot urine-Tanaka:<br>Total: 3356.68 ± 860. 28<br>Females: 3494.49 ± 951.23<br>Males: 3209.36 ± 739.25<br><br>Based on 24-hr urine:<br>Total: 3555.42 ± 1654.13<br>Males: 4092.89 ± 1735.92

					Females: 2980.88 ± 1369.35
Helou 2014 – Thesis [42]<br><br>**Cross-sectional; convenience sampling**	--	Regional	Spot urine with urinary creatinine + FFQ and 24-hr recalls	**Adults aged 19-55 years;** N=100	***Mean Na intake (mg/day):***<br>Based on FFQ:<br>Total: 4618.26 ± 1898.02<br>Males: 5237.09 ± 1894.06<br>Females: 4091.26 ± 1690.27<br><br>Based on 24-hr recall:<br>Total: 3395.04 ± 1282.90<br>Males: 3786.11 ± 1387.24<br>Females: 3124.10 ± 1143.77<br><br>Based on urine:<br>Total: 4573.20 ± 1372.69<br>Males: 5014.44 ± 1456.71<br>Females: 4255.52 ± 1243.13
Hamamji 2018-Thesis [43]<br><br>**Cross-sectional surveys; stratified cluster sampling**	2012 underfive; 2014 the other one	National	24-hr recall	**Underfive children and children and adolescents aged 6-18 years;**<br>N=888 underfive and 1106 children and adolescents	***Mean Na intake (g/day):***<br>-Underfive: 0.9-1.7<br>-Children and adolescents: 1.9-2.3<br><br>6 months-2 years:<br>Total: 0.87 ± 0.03<br>Boys: 0.9 ± 0.05<br>Girls: 0.84 ± 0.05<br><br>3-5 year olds:<br>Total: 1.73 ± 0.04<br>Boys: 1.78 ± 0.05<br>Girls: 1.67 ± 0.06<br><br>6-8 year olds:<br>Total: 1.95 ± 0.06

						Boys: 2.07 ± 0.1<br>Girls: 1.84 ± 0.08<br><br><u>9-13 year olds:</u><br>Total: 2.31 ± 0.06<br>Boys: 2.45 ± 0.08<br>Girls: 2.16 ± 0.08<br><br><u>14-18 year olds:</u><br>Total: 2.21 ± 0.09<br>Boys: 2.75 ± 0.15<br>Girls: 1.84 ± 0.1
	Aoun et al 2019 [44]<br><br>**Cross-sectional; random household sampling method**	2017	National	FFQ and 24-hr recalls	**Adults aged 18-60 years;**<br>N=114	***Mean Na intake (mg/day):***<br><u>Based on FFQ:</u><br>4308.24 ± 756.53<br><br><u>Based on 24-hr recalls:</u><br>4412.26 ± 767.07
	Almedawar et al 2015 [45]	2008-2009	National	Dietary diary	**Adults aged 20 years and above;**<br>N=2543	***Mean Na intake (g/day):***<br>Total: 2.9<br>Males: 3.4<br>Females: 2.4
**Morocco**	MOH [46]	2017-2018	National	Spot urine	**Adults aged 18 years and above**	***Mean salt intake (g/day):***<br>Total: 10.6 (10.5-10.7)<br>Males: 11.9 (11.7-12.0)<br>Females: 9.3 (9.2-9.4)<br><br><u>30-44 year olds:</u><br>11.1 (11.0-11.3)<br><u>45-59 year olds:</u><br>10.9 (10.7-11.0)

	Saeid et al 2018 [47]<br><br>**Transversal**	2015-2016	Regional; Rabat and its region	24-hr Na urinary excretion + urinary creatinine	**School-aged children 6-18 years** from schools;<br>N=131	***Mean Na intake (mg/day):***<br>Total: 2235.3 ± 823.2 (equivalent to 5667.9 ± 2077.7 mg/day of salt)<br>Boys: 2184.3 ± 783.3<br>(equivalent to 5548.3 ± 1989.6 mg/day of salt)<br>Girls: 2290.3 ± 867.2<br>(equivalent to 5797 ± 2177.5 mg/day of salt)
	Derouiche et al 2016 and Derouiche et al 2017 [48,49]<br><br>**Pilot study**	2014	Regional; Central	24-hr Na urinary excretion + urinary creatinine	**Adults aged 24-64 years;**<br>N=128	***Mean Na intake (mg/day):***<br>Total: 2779.1 ± 1334.9<br>Males: 2927.6 ± 1227.5<br>Females: 2640.3 ± 1427.6
	Anzid et al 2014 [50]<br><br>**Self-selected sample**	2007-2008	Regional; Semi-urban, Ouarzazate	Food records	**High school students (15-19 years)** from 5 public high schools;<br>N=293	***Mean Na intake (mg/day):***<br>Total: 2580 ± 586.1<br>Boys: 2847.7 ± 578.3<br>Girls: 2399.4 ± 519.8
**Oman**	Al-Mawali et al 2020 [51]<br><br>**Cross-sectional population-based survey**	2017-2018	National	24-hr Na urinary excretion + urinary creatinine	**Adults aged 18 years and above;**<br>N=569	***Mean salt intake (g/day):***<br>Total: 9<br>Males: 9.6<br>Females: 8.7
	Al-Mawali et al 2020 [52]<br><br>**Cross-sectional; based on the WHO STEPs**	2017	National	Spot urine	**Adults aged 18 years and above, as nationals and non-Omani resident;**<br>9053 households	***Mean salt intake (g/day):***<br><u>Total sample:</u><br>Total: 8.6<br>Males: 9.5<br>Females: 7.4<br><br><u>Amongst Omani citizens only:</u><br>Total: 8.4<br>Males: 9.6<br>Females: 7.4

	Al-Ghannami 2004 [53]	--	National	24-hr recall	**National Nutrition Survey**	***Mean salt intake (g/day):***<br>11-12
	MOH [54]	2017	National	NCDs National Survey	**--**	***Mean salt intake (g/day):***<br>Total: 8.5<br>Males: 9.6<br>Females: 7.5
**Pakistan**	Saqib et al 2020 [55]<br><br>**Descriptive cross-sectional; non-probability convenient sampling**	--	Regional; Islamabad	24-hr Na urinary excretion + urinary creatinine + spot urines	**Adults aged 18 years and above**;<br>N=120	***Mean salt intake (g/day):***<br>8.64 ± 4.43
	Rifat uz Zaman et al 2013 [56]<br><br>**Cross-sectional; descriptive; convenience sample**	--	Regional; Urban Sialkot	3-day food record	**High school students;**<br>N=328	***Mean Na intake (mg/day):***<br>Total: 1173.5 ± 46.3<br>Males: 1224.6 ± 50.6<br>Females: 1058.4 ± 44.5
**Palestine**	ElMadfa et al 2014 [57]	2013	National	Spontaneous urine	**Schoolchildren aged 7-12 years**	***Mean salt intake (g/day):***<br>7 (6.4 ± 4.2 in the West Bank and 7.3 ± 4.4 in the Gaza Strip)
**Sudan**	MOH-WHO [58]<br><br>**STEPs survey**	2016	National	Spot urine	**Adults aged 18 to 69 years**	***Mean salt intake (g/day):***<br>Total: 8.2<br>Males: 8.2<br>Females: 8.2
**Tunisia**	Doggui et al 2021 [59]<br><br>**Cross-sectional; multistage**	2015	Regional; Urban region, Bizerte	24-hr Na urinary excretion + urinary creatinine	**Adults aged 24-64 years**;<br>N=194	***Mean salt intake (g/day):***<br>8.1 ± 2.7
	WHO technical report on the EMR in Tunisia 2015 [60]	--	National	24-hr recall	**Entire population**	***Mean Na intake (g/day):***<br>Total: 10.2<br>Males: 11.3

						Females: 10
	Aounallah-Skhiri et al 2011 [61]<br><br>**Cross-sectional; clustered random sample**	2005	Regional; 3 regions	Semi-quantitative FFQ	**Adolescents aged 15-19 years;**<br>N=1019	***Mean Na intake (mg/day):***<br>Total: 1648.9 ± 9.9<br>Males: 1641.5 ± 13.3<br>Females: 1656.5 ± 10.2
	National Survey 2016 [62]	2016	National	National survey	**15 year olds and above**	***Mean salt intake (g/day):***<br>10-12
**UAE**	Jarrar et al 2020 [63]<br><br>**Cross-sectional**	2015	National	24-hr Na urinary excretion + urinary creatinine	**Adults aged 20-65 years;**<br>N=477	***Mean Na intake (mg/day):***<br>2713.4 ± 713
	Cheikh Ismail 2019 [64]	2018	Regional; University of Sharjah	24-hr recall	**Students**	***Mean Na intake (mg/day):***<br>Males: 3677<br>Females: 3464

Abbreviations: EMR: Eastern Mediterranean Region; FFQ: food frequency questionnaire; KSA: Kingdom of Saudi Arabia; MAP: Multisectoral Action Plan; MOH: Ministry of Health; MOHAP: Ministry of Health and Prevention; MOHME: Ministry of Health and Medical Education; Na: sodium; NCD: non-communicable disease; UAE: United Arab Emirates; WHO: World Health Organization.

References

1. Abdul Wahab, A.; Moosa, K.; Gharib, N.; Al-Sairafi, M.; Al-Raees, G.; Al-Amer, M. National nutrition survey for adult Bahrainis aged 19 years and above; Ministry of Health: Kingdom of Bahrain, 2002. https://www.semanticscholar.org/paper/NATIONAL-NUTRITION-SURVEY-FOR-ADULT-BAHRAINIS-AGED-Wahab-Sairafi/b5cfd2ef26840b94a1f317300303f5d389c5009e.
2. Central Agency for Public Mobilization and Statistics-Ministry of Health and Prevention-World Health Organization. Egypt National STEPwise Survey For Noncommunicable Diseases Risk Factors Report 2017; 2018. https://www.who.int/ncds/surveillance/steps/Egypt_STEPS_Survey_2017_Fact_Sheet.pdf?ua=1.
3. AbdEl Aal, A.; Abdul-Aziz, A.; Zaghloul, S. Construct validity of 24 hour recall estimating accuracy of energy and sodium intake. *Egypt J Nutr* **2018**, *33*, 1-28.
4. Brouzes, C.M.C.; Darcel, N.; Tomé, D.; Dao, M.C.; Bourdet-Sicard, R.; Holmes, B.A.; Lluch, A. Urban Egyptian women aged 19–30 years display nutrition transition-like dietary patterns, with high energy and sodium intakes, and insufficient iron, vitamin D, and folate intakes. *Curr Dev Nutr* **2020**, *4*, doi:10.1093/cdn/nzz143.
5. Zaghloul, S.; Mogeid, A.-E.; ElAal, A.; Fattah, N. Sources of dietary sodium among Egyptian adults. *Egypt J Nutr* **2018**, *3*, 81-91.

6. Tayel, D.I.; Amine, A.K.; El-Amina, K. Dietary intake of nutrients related to bone health among Alexandria University female students, Egypt. *Food Public Health* **2013**, *3*, 329-335.
7. World Health Organization Global Database on the Implementation of Nutrition Action (GINA). Policy - Egypt National Multisectoral Action Plan for Prevention and Control of Noncommunicable Diseases (EgyptMAP-NCD) - Egypt; 2017. https://extranet.who.int/nutrition/gina/en/node/25915.
8. Gholami, A.; Rezaei, S.; Jahromi, L.M.; Baradaran, H.R.; Ghanbari, A.; Djalalinia, S.; Rezaei, N.; Naderimagham, S.; Modirian, M.; Mahmoudi, N., et al. Is salt intake reduction a universal intervention for both normotensive and hypertensive people: a case from Iran STEPS survey 2016. *Eur J Nutr* **2020**, *59*, 3149-3161, doi:10.1007/s00394-019-02153-8.
9. Rezaei, S.; Mahmoudi, Z.; Sheidaei, A.; Aryan, Z.; Mahmoudi, N.; Gohari, K.; Yoosefi, M.; Hajipour, M.J.; Dilmaghani-Marand, A.; Soleimanzadehkhayat, M., et al. Salt intake among Iranian population: The first national report on salt intake in Iran. *J Hypertens* **2018**, *36*, 2380-2389, doi:10.1097/HJH.0000000000001836.
10. Ministry of Health and Medical Education-Tehran University of Medical Sciences-National Institute of Health Research-Iran. Atlas of Non-Communicable Diseases Risk- Factors Surviellance in the Islamic Republic of Iran- STEPS 2016; 2016. https://www.who.int/ncds/surveillance/steps/STEPS_2016_Atlas_EN.pdf?ua=1.
11. Khosravi, A.; Kelishadi, R.; Sarrafzadegan, N.; Boshtam, M.; Nouri, F.; Zarfeshani, S.; Esmaillzadeh, A. Impact of a community-based lifestyle intervention program on blood pressure and salt intake of normotensive adult population in a developing country. *Journal of research in medical sciences : the official journal of Isfahan University of Medical Sciences* **2012**, *17*, 1-7.
12. Khosravi, A.; Toghianifar, N.; Sarrafzadegan, N.; Gharipour, M.; Azadbakht, L. Salt intake, obesity, and pre-hypertension among iranian adults: A cross-sectional study. *Pak J Med Sci* **2012**, *28*, 297-302.
13. Arsang-Jang, S.; Mansourian, M.; Mohammadifard, N.; Khosravi, A.; Oveis-Gharan, S.; Nouri, F.; Sarrafzadegan, N. Temporal trend analysis of stroke and salt intake: a 15-year population-based study. *Nutr Neurosci* **2019**, 10.1080/1028415X.2019.1638665, doi:10.1080/1028415X.2019.1638665.
14. Mohammadifard, N.; Khosravi, A.; Salas-Salvadó, J.; Becerra-Tomás, N.; Nouri, F.; Abdollahi, Z.; Jozan, M.; Bahonar, A.; Sarrafzadegan, N. Trend of salt intake measured by 24-hour urine collection samples among Iranian adults population between 1998 and 2013: The Isfahan salt study. *Nutr Metabo Cardiovasc Dis* **2019**, *29*, 1323-1329, doi:10.1016/j.numecd.2019.07.019.
15. Motlagh, Z.; Mazloomy, S.; Mozaffari Khosravi, H.; Morowatisharifabad, M.; Askarshahi, M. Salt intake among women refer to medical health centers, Yazd, Iran, 2011. *SSU_Journals* **2011**, *19*, 550-560.
16. Mirzaei, M.; Soltani, M.; Namayandeh, M.; GharahiGhehi, N. Sodium and potassium intake of urban dwellers: Nothing changed in Yazd, Iran. *Journal of health, population, and nutrition* **2014**, *32*, 111-117.
17. Emamian, M.H.; Ebrahimi, H.; Hashemi, H.; Fotouhi, A. Salt intake and blood pressure in Iranian children and adolescents: a population-based study. *BMC Cardiovasc Disord* **2021**, *21*, doi:10.1186/s12872-021-01876-z.
18. Kelishadi, R.; Gheisari, A.; Zare, N.; Farajian, S.; Shariatinejad, K. Salt intake and the association with blood pressure in young Iranian children: First report from the middle east and North Africa. *Int J Prev Med* **2013**, *4*, 475-483.
19. Mohammadifard, N.; Khaledifar, A.; Khosravi, A.; Nouri, F.; Pourmoghadas, A.; Feizi, A.; Esmaillzadeh, A.; Sarrafzadegan, N. Dietary sodium and potassium intake and their association with blood pressure in a non-hypertensive Iranian adult population: Isfahan salt study. *Nutrition & dietetics: the journal of the Dietitians Association of Australia* **2017**, *74*, 275-282, doi:10.1111/1747-0080.12304.

20. Rafiei, M.; Boshtam, M.; Sarraf-Zadegan, N.; Seirafian, S. The relation between salt intake and blood pressure among Iranians. *Kuwait Med J* **2008**, *40*, 191-195.
21. Karimi, G.; Heidari-Beni, M.; Riahi, R.; Qorbani, M.; Kelishadi, R. Healthy eating index in a nationally representative sample of children and adolescents by socio-demographic characteristics: the Weight disorders survey of the CASPIAN-IV Study. *Turk J Pediatr* **2020**, *62*, 930-939, doi:10.24953/turkjped.2020.06.004.
22. Mirmiran, P.; Ziadlou, M.; Karimi, S.; Hosseini-Esfahani, F.; Azizi, F. The association of dietary patterns and adherence to WHO healthy diet with metabolic syndrome in children and adolescents: Tehran lipid and glucose study. *BMC Public Health* **2019**, *19*, doi:10.1186/s12889-019-7779-9.
23. Shahriarpour, Z.; Nasrabadi, B.; Shariati-Bafghi, S.E.; Karamati, M.; Rashidkhani, B. Adherence to the dietary approaches to stop hypertension (DASH) dietary pattern and osteoporosis risk in postmenopausal Iranian women. *Osteoporosis international : a journal established as result of cooperation between the European Foundation for Osteoporosis and the National Osteoporosis Foundation of the USA* **2020**, *31*, 2179-2188, doi:10.1007/s00198-020-05450-9.
24. Azizi, F.; Rahmani, M.; Allahverdian, S.; Hedayati, M. Effects of salted food consumption on urinary iodine and thyroid function tests in two provinces in the Islamic Republic of Iran. *East Mediterr Health J* **2001**, *7*, 115-120.
25. Rahmani, M.; Koohkan, A.; Allahverdian, S.; Hedayati, M. Comparison of dietary iodine intake and Urinary excretion in urban and rural Households of Ilam in 2000. *Iran J Endocrinol Metab* **2000**, *2*, 31-37.
26. Kooshki, A.; Golafrooz, M. Nutrient intakes affecting bone formation compared with dietary reference intake (DRI) in Sabzevar elderly subjects. *Pak J Nutr* **2009**, *8*, 218-221, doi:10.3923/pjn.2009.218.221.
27. Ministry of Health-World Health Organization. Noncommunicable Diseases Risk Factors STEPS Survey Iraq 2015; 2015. https://www.who.int/ncds/surveillance/steps/Iraq_2015_STEPS_Report.pdf?ua=1.
28. Alawwa, I.; Dagash, R.; Saleh, A.; Ahmad, A. Dietary salt consumption and the knowledge, attitudes and behavior of healthy adults: a cross-sectional study from Jordan. *The Libyan journal of medicine* **2018**, *13*, doi:10.1080/19932820.2018.1479602.
29. Ministry of Health-Jordan. Jordan National Stepwise Survey (STEPs) for Noncommunicable Diseases Risk Factors 2019; 2020. https://www.moh.gov.jo/Echobusv3.0/SystemAssets/42e9a4d5-b719-4047-9fb6-c2b60adfee3a.pdf.
30. Al-Wa'l, T.; Takruri, H. Sodium and potassium intakes in a sample of students and employees in the University of Jordan aged (20-40 years) using 3-day food diaries. *Nutr Food Sci* **2016**, *46*, 43-50, doi:10.1108/NFS-06-2015-0067.
31. Alkurd, R.A. Estimated intakes of fats, cholesterol, fiber, sodium, calcium, potassium, and magnesium in Jordan. *Aust J Basic Appl Sci* **2011**, *5*, 3171-3178.
32. Takruri, H.R.; Alkurd, R.A. Intakes of Fats, Cholesterol, Fiber and Micronutrients as Risk Factors for Cardiovascular Disease in Jordan. *Jordan J Biol Sci* **2014**, *7*.
33. Alkhunaizi, A.M.; Al, J.H.; Al, S.Z. Salt intake in Eastern Saudi Arabia. *East Mediterr Health J* **2013**, *19*, 915-918.
34. Al-Khathaami, A.; Roaa, A.; Sara, Q.; Azhar, A.; Bashayer, A.; Alaa, A. Evaluation of dietary salt intake among healthy students in Riyadh, Saudi Arabia. *J Health Inform Dev Ctries* **2019**, *13*, unpaginated.
35. Zaghloul, S.; Al-Hooti, S.N.; Al-Hamad, N.; Al-Zenki, S.; Alomirah, H.; Alayan, I.; Al-Attar, H.; Al-Othman, A.; Al-Shami, E.; Al-Somaie, M. Evidence for nutrition transition in Kuwait: over-consumption of macronutrients and obesity. *Public Health Nutr* **2013**, *16*, 596-607.
36. Alomirah, H.; Al-Zenki, S.; Husain, A.; al., e. Assessment of Acrylamide Levels in Heat-Processed Foodstuffs Consumed by Kuwaitis. *Library of the Kuwait Institute of Scientific Research and Essays* **2008**, *KISR No. 9316*.
37. The Public Authority for Food and Nutrition-Kuwait. The Case for Investment in Prevention and Control of Non-Communicable Diseases in Kuwait; 2020.

38. World Health Organization. Kuwaitis lower blood pressure by reducing salt in bread. Availabe online: https://www.who.int/features/2014/kuwait-blood-pressure/en/#:~:text=But%20in%20Kuwait%2C%20cutting%20the,most%20popular%20type%20of%20bread (accessed on 4 March 2021).
39. Merhi, K.A. Urinary sodium and potassium status of Lebanese school aged children. American University of Beirut, Beirut, Lebanon, 2017.
40. Nasreddine, L.; Hwalla, N.; Ismaeel, H. Validation of a food frequency questionnaire for the assessment of sodium dietary intake using 24-hour urine sodium excretion in Lebanese adults. 2014. *American University of Beirut (unpublished)* **2014**.
41. Chouccair, S.R. Validation of a food frequency questionnaire and a spot urine sample for the assessment of dietary sodium intake in Lebanese adults. American University of Beirut, Beirut, Lebanon, 2016.
42. Helou, R.M. Development and validation of a food frequency questionnaire for the assessment of sodium dietary intake in Lebanese adults. American University of Beirut, Beirut, Lebanon, 2014.
43. Hamamji, S.E. Intakes and sources of fat, free sugars and salt among Lebanese children and adolescents. American University of Beirut, Beirut, Lebanon, 2018.
44. Aoun, C.; Daher, R.B.; Osta, N.E.; Papazian, T.; Khabbaz, L.R. Reproducibility and relative validity of a food frequency questionnaire to assess dietary intake of adults living in a Mediterranean country. *PLoS ONE* **2019**, *14*, doi:10.1371/journal.pone.0218541.
45. Almedawar, M.M.; Nasreddine, L.; Olabi, A.; Hamade, H.; Awad, E.; Toufeili, I.; Arnaout, S.; Isma'eel, H.A. Sodium intake reduction efforts in Lebanon. *Cardiovasc Diagn Ther* **2015**, *5*, 178-185.
46. Ministry of Health-Morocco. National Survey on Common Risk Factors for Non- Communicable Diseases 2017-2018; 2018. https://www.sante.gov.ma/Publications/Etudes_enquete/Pages/default.aspx.
47. Saeid, N.; Elmzibri, M.; Hamrani, A.; Latifa, Q.; Belghiti, H.; El Berri, H.; Benjeddou, K.; Bouziani, A.; Benkirane, H.; Taboz, Y., et al. Assessment of Sodium and Potassium Intakes in Children Aged 6 to 18 Years by 24 h Urinary Excretion in City of Rabat, Morocco. *J Nutr Metab* **2018**, *2018*, doi:10.1155/2018/8687192.
48. Derouiche, A.; El-Kardi, Y.; Mohtadi, K.; Jafri, A. Estimation of the Daily Salt Intake by 24-Hour Urinary Sodium Excretion in Morocco: A Pilot Study. **2016**.
49. Derouiche, A.; El-kardi, Y.; Mohtadi, K.; Jafri, A. Salt intake assessed by 24 hour urinary sodium excretion of Moroccan adults: A pilot study. *Nutr Clin Metab* **2017**, *31*, 207-211, doi:10.1016/j.nupar.2017.07.001.
50. Anzid, K.; Baali, A.; Vimard, P.; Levy-Desroches, S.; Cherkaoui, M.; López, P.M. Inadequacy of vitamins and minerals among high-school pupils in Ouarzazate, Morocco. *Public Health Nutr* **2014**, *17*, 1786-1795, doi:10.1017/S1368980013002140.
51. Al-Mawali, A.; D'Elia, L.; Jayapal, S.K.; Morsi, M.; Al-Shekaili, W.N.; Pinto, A.D.; Al-Kharusi, H.; Al-Balushi, Z.; Idikula, J.; Al-Harrasi, A., et al. National survey to estimate sodium and potassium intake and knowledge attitudes and behaviours towards salt consumption of adults in the Sultanate of Oman. *BMJ Open* **2020**, *10*, doi:10.1136/bmjopen-2020-037012.
52. Al-Mawali, A.; Jayapal, S.K.; Morsi, M.; Al-Shekaili, W.; Pinto, A.D.; Al-Kharusi, H.; Al-Harrasi, A.; Al-Balushi, Z.; Idikula, J. Prevalence of Risk Factors of Non-Communicable Diseases in the Sultanate of Oman: STEPS Survey 2017. **2020**.
53. Al-Ghannami, S. National Nutrition Survey; Library of Ministry of Health: Ministry of Health. Sultanate of Oman, 2004.
54. Ministry of Health-Oman. MOH Announces NCDs National Survey Results; 2018. https://www.moh.gov.om/en/-/---896.
55. Saqib, M.A.N.; Rafique, I.; Ansar, M.; Rahat, T. Daily Salt Intake, its Discretionary Use and Validation of Methods for Estimation using Spot Urine, Findings from Islamabad, Pakistan. *medRxiv* **2020**.

56. Rifat uz, Z.; Iqbal, Z.; Ali, U. Dietary intakes of urban adolescents of Sialkot, Pakistan do not meet the standards of adequacy. *Pak J Nutr* **2013**, *12*, 460-467, doi:10.3923/pjn.2013.460.467.
57. Elmadfa, A.; Ben-Abdullah, K.; Meyer, A.; Ramlawi, A.; Bahar, L.; Rizkallah, N.; Skaik, A.; Zakout, Z.; El-Aissaw, F. Palestine Micronutrient Survey 2013; UNICEF, University of Vienna: 2014.
58. Ministry of Health-World Health Organization. Sudan STEPwise Survey for Non-Communicable Disease Risk Factors 2016 Report; 2016. https://www.who.int/ncds/surveillance/steps/Sudan_STEPwise_SURVEY_final_2016.pdf?ua=1.
59. Doggui, R.; El Ati, J.; Sassi, S.; Ben Gharbia, H.; Al-Jawaldeh, A.; El Ati-Hellal, M. Unbalanced intakes of sodium and potassium among Tunisian adults: A cross-sectional study. *Food Sci Nutr* **2021**, 10.1002/fsn3.2197, doi:10.1002/fsn3.2197.
60. World Health Organization. Report on the technical consultation on salt and fat reduction strategies in the Eastern Mediterranean Region, Tunis, Tunisia 30–31 March 2015; World Health Organization. Regional Office for the Eastern Mediterranean: 2015.
61. Aounallah-Skhiri, H.; Traissac, P.; El Ati, J.; Eymard-Duvernay, S.; Landais, E.; Achour, N.; Delpeuch, F.; Romdhane, H.B.; Maire, B. Nutrition transition among adolescents of a south-Mediterranean country: dietary patterns, association with socio-economic factors, overweight and blood pressure. A cross-sectional study in Tunisia. *Nutr J* **2011**, *10*, 1-17.
62. World Health Organization Global Database on the Implementation of Nutrition Action (GINA). Policy - Stratégie Nationale Multisectorielle de Prévention et Contrôle des Maladies Non Transmissibles (MNT) - Tunisia; 2018. https://extranet.who.int/nutrition/gina/en/node/39424.
63. Jarrar, A.H.; Stojanovska, L.; Apostolopoulos, V.; Ismail, L.C.; Feehan, J.; Ohuma, E.O.; Ahmad, A.Z.; Alnoaimi, A.A.; Al Khaili, L.S.; Allowch, N.H., et al. Assessment of sodium knowledge and urinary sodium excretion among regions of the united arab emirates: A cross-sectional study. *Nutrients* **2020**, *12*, 1-14, doi:10.3390/nu12092747.
64. Cheikh Ismail, L.; Hashim, M.; H Jarrar, A.; N Mohamad, M.; T Saleh, S.; Jawish, N.; Bekdache, M.; Albaghli, H.; Kdsi, D.; Aldarweesh, D. Knowledge, attitude, and practice on salt and assessment of dietary salt and fat intake among University of Sharjah students. *Nutrients* **2019**, *11*, 941.
